# Prevalence of Candidate Vaccine Targets and Genomic Features of Pediatric Invasive *Streptococcus Agalactiae* in Japan

**DOI:** 10.1093/infdis/jiaf491

**Published:** 2025-10-09

**Authors:** Masashi Kasai, Satoshi Nakano, Shota Koide, Shogo Otake, Meiwa Shibata, Kasumi Ishida-Kuroki, Yo Sugawara, Yukihiro Akeda, Kandai Nozu, Motoyuki Sugai

**Affiliations:** Division of Infectious Disease, Hyogo Prefectural Kobe Children's Hospital, Kobe, Japan; Department of Pediatrics, Kobe University Graduate School of Medicine, Kobe, Japan; Antimicrobial Resistance Research Center, National Institute of Infectious Diseases, Tokyo, Japan; Department of Bacteriology I, National Institute of Infectious Diseases, Toyama, Tokyo, Japan; Antimicrobial Resistance Research Center, National Institute of Infectious Diseases, Tokyo, Japan; Department of Pediatrics, Kobe University Graduate School of Medicine, Kobe, Japan; Antimicrobial Resistance Research Center, National Institute of Infectious Diseases, Tokyo, Japan; Division of Infectious Diseases, Department of Pediatrics, Tokyo Metropolitan Children's Medical Center, Tokyo, Japan; Center for Pediatric Infectious Diseases, Tokyo Metropolitan Children's Medical Center, Tokyo, Japan; Antimicrobial Resistance Research Center, National Institute of Infectious Diseases, Tokyo, Japan; Antimicrobial Resistance Research Center, National Institute of Infectious Diseases, Tokyo, Japan; Department of Bacteriology I, National Institute of Infectious Diseases, Toyama, Tokyo, Japan; Department of Pediatrics, Kobe University Graduate School of Medicine, Kobe, Japan; Antimicrobial Resistance Research Center, National Institute of Infectious Diseases, Tokyo, Japan

**Keywords:** Group B *Streptococcus*, S*treptococcus agalactiae*, transmission, vaccine, pediatric

## Abstract

**Background:**

*Streptococcus agalactiae* (Group B *Streptococcus*, GBS) is a leading cause of invasive neonatal and infant infections, including sepsis and meningitis. This study aimed to estimate the vaccine coverage and characterize the genomic features of pediatric invasive GBS in Japan.

**Methods:**

We conducted a nationwide, multicenter, retrospective genomic surveillance study involving 237 GBS isolates from sterile specimens of children aged ≤15 years across 35 hospitals in Japan between 2004 and 2023. Serotyping, antimicrobial susceptibility testing, and whole-genome sequencing were performed.

**Results:**

The estimated vaccine coverage was 98.3% for the hexavalent polysaccharide vaccine and 94.9% for the GBS-NN/NN2 protein vaccine. Erythromycin and clindamycin resistance were observed in 61.2% and 43.5%, respectively. Among the 75 CC17 isolates, 59 (78.7%) contained only PI-2B and harbored both *ermB* and *tetO*, indicating the predominance of a multidrug-resistant clone. Single nucleotide polymorphism–based analysis revealed evidence of nosocomial transmission and persistent regional circulation, particularly within the ST17 and ST23 lineages.

**Conclusions:**

This study suggests that current maternal GBS vaccine candidates would provide broad coverage for pediatric invasive infections in Japan. The identification of persistent and regionally disseminated lineages highlights the importance of investigating potential, yet, poorly understood, transmission routes, including environmental reservoirs, to inform future prevention strategies.

## Teaser

Nationwide genomic surveillance of pediatric invasive Group B Streptococcus in Japan (2004–2023) showed vaccine coverage of 98.3% (hexavalent polysaccharide) and 94.9% (GBS-NN/NN2), with SNP analysis revealing long-term regional circulation of ST17 and ST23 clones.

*Streptococcus agalactiae*, also known as Lancefield Group B *Streptococcus* (GBS), causes life-threatening neonatal infections, such as sepsis and meningitis [1]. GBS infections in children are classified as early-onset disease (EOD; ≤ 6 days), late-onset disease (LOD; 7–89 days), and very late-onset disease (VLOD; ≥ 90 days) [2].

Group B Streptococcus infections result in a considerable disease burden worldwide. Over 390 000 newborns are affected annually, resulting in 46 200 stillbirths, 91 900 deaths, and 37 100 neurodevelopmentally impaired survivors [3]. The disease burden varies globally, with the majority occurring in low- and middle-income countries (LMICs). Sub-Saharan Africa has the highest rates, accounting for nearly half of all GBS-related deaths worldwide [3].

Vertical transmission of colonized GBS from the mother is the primary risk factor for invasive GBS infections in neonates [4]. To prevent EOD, numerous countries have adopted intrapartum antibiotic prophylaxis (IAP) using either risk-based or screening-based approaches [5]. However, although the introduction of IAP has dramatically reduced the occurrence of EOD, LOD is still reported worldwide [6]. One possible explanation is the unclear route of transmission to newborns and infants [6]. Berardi et al. [7] retrospectively studied 98 cases of LOD in Italy, evaluating maternal GBS colonization status and serotypes in relation to the isolates from the infected infants. They found that approximately 44% of mothers were colonized with GBS at the vaginal/rectal site during prenatal screening, and approximately 66% were colonized by the time of disease onset. In approximately one-third of LOD cases, the infection source remains unidentified. These results suggest the possibility of transmission routes other than the mother, such as caregiver contact [6] or exposure to contaminated food or animals [8, 9]. Furthermore, the high rate of resistance to clindamycin (CLI), a second-line antibiotic for IAP, is a concern [10].

To decrease the burden of invasive GBS infections in newborns and infants, the World Health Organization (WHO) set milestones to ensure that at least one affordable GBS vaccine is licensed and WHO-prequalified for maternal immunization during pregnancy by 2028 and that it is introduced in at least 10 countries by 2030 [11]. Maternal vaccines are classified into 2 categories: polysaccharide and protein-based vaccines. The former targets the 3 or 6 most prevalent capsular polysaccharide (CPS) serotypes associated with invasive infections among the 10 serotypes (Ia, Ib, II–IX) [12]. In a phase II trial, the hexavalent vaccine elicited anti-CPS antibodies in pregnant women, which were then transferred to infants [4]; however, it remains unclear whether these transplacentally transferred antibodies are effective in preventing invasive GBS infections in infants, and concerns remain regarding serotype replacement or capsular switching [13]. Although various proteins, including pili [14] and serine-rich repeat (srr) proteins [15], have been explored as protein-based vaccine targets [13], current clinical trials are focusing on 4 members of the alpha-like protein (Alp) family: Alpha C, Alp1, Alp2/3, and Rib. These proteins are conserved across nearly all GBS strains [16] and have considerable structural similarities [17]. A phase I trial of the GBS-NN vaccine targeting the N-terminal domains of Alpha C and Rib demonstrated good safety and immunogenicity; however, heterotypic response against other members of Alp family varied among subjects, depending on homologous pre-existing immunity [17]. Consequently, a phase I trial of a second-generation vaccine (GBS-NN/NN2), which targets the N-terminal domains of all 4 members, was conducted: the results demonstrated robust and persistent antibody response [18]. These vaccines are administered to pregnant women to induce antibody production, which is transferred across the placenta, providing passive immunity to the infant.

Based on this background, this study estimated the coverage rates of each potential GBS vaccine. Furthermore, through whole-genome analysis, we analyzed the genomic features of GBS isolates in Japan, including resistance genes, and elucidated the genetic relationships among isolates detected independently at different times and geographic regions.

## METHODS

### Study Design and Period

We conducted a retrospective genomic surveillance study at 167 hospitals in Japan, including 18 children's hospitals and 32 university hospitals. Our study included patients aged 15 years or younger from whom GBS was isolated from sterile specimens (eg, blood, cerebrospinal fluid, and joint fluid) before May 2023. Patients whose specimens were not preserved at the hospital were excluded.

### Data Collection

Hospitals provided both isolates and corresponding clinical information (birth date, sampling date, sex, and type of sterile specimen), along with geographic data. Although the age of onset was unavailable, we assumed that the onset and sampling dates were close in severe infant infections. Therefore, we classified the disease type based on age at the time of specimen collection.

When GBS was detected in multiple sterile specimens, the infection site sample was prioritized to avoid sample duplication. For example, when GBS was detected in the blood and cerebrospinal fluid (or joint fluid), data from the latter were prioritized. If GBS was detected again in the same patient >28 days after the previous detection, it was considered a different isolate.

## ANALYSIS OF *S. AGALACTIAE* ISOLATES, SEROTYPING, SUSCEPTIBILITY TEST, AND WHOLE-GENOME SEQUENCE

### Serotyping

We serotyped all isolates using the GBS latex agglutination test kit, The ImmuLex™ Strep-B Kit (SSI Diagnostica, Denmark), according to the manufacturer's instructions. Isolates with no agglutination were classified as non-typeable.

### Susceptibility Test

Antimicrobial susceptibility tests were performed using the broth microdilution method, following the Clinical and Laboratory Standards Institute (CLSI) reference standards 2023 [19]. We determined the minimum inhibitory concentrations of penicillin (PEN), cefotaxime (CTX), erythromycin (ERY), CLI, levofloxacin (LVX), and tetracycline (TET). In addition, we assessed inducible CLI resistance using wells containing a combination of 0.5 μg/mL CLI and 1 μg/mL ERY [19].

The categories of susceptible (S), intermediate resistance (I), and resistant (R) were based on CLSI guidelines. The CLSI does not define the categories of I or R; however, in this study, we defined isolates that were not categorized as “S” as “R” to improve readability.

### Whole-genome Sequencing Analysis

We obtained 150 bp paired-end short-reads using Illumina NovaSeq X Plus platform. After trimming and quality checks, we analyzed the data using an in-house pipeline [20]. We detected the following candidate vaccine targets according to previous studies: hypervirulent GBS adhesin [HvgA], Srr proteins [Srr1 and Srr2], members of Alp family [AlphaC, Alp1, Alp2/3, and Rib], pilus islands [PI-1, PI-2A, and PI-2B], BibA, C5a peptidase, enolase, glyceraldehyde-3-phosphate dehydrogenase, gbs2106, LrrG, SIP, SAN_0226, SAN_0356, SAN_0413, SAN_0990, SAN_1040, SAN_1577, SAN_1685 and SAN_1808 [16, 21–23].

### Phylogenetic Tree Reconstruction and Estimating Recombinational Events

First, we constructed a maximum-likelihood tree for all tested isolates using RAxML Next Generation v1.1 [24] followed by BAPS clustering using rhierBAPS v1.0.1 [25]. We then estimated the recombination regions and event frequencies using Gubbins v3.4 [26] for ST10, ST17, ST23, and ST335, with >10 isolates in each ST.

### Dating the Origin of ST10, ST17, and ST23 in Japan

The root dates of ST10, ST17, and ST23, with ≥20 isolates, were analyzed using the Bayesian Markov chain Monte Carlo framework by BEAST2 [27] with a clock rate estimation. To generate input files for BEAST analysis, we first mapped the trimmed reads to a reference sequence of each ST (Supplementary Table 1) followed by masking of recombination sites that were predicted by Gubbins. Lastly, we masked mobile genetic element sites that were predicted in each reference sequence by geNomad [28]. The significance of the temporal signals of the dataset was measured by a permutation test using Bactdating v1.1 [29].

### SNP Analysis and the Detection of Potential Endemic GBS Subclusters

To measure the number of SNPs between isolates and to understand the relationship between the number of SNPs, the region of isolation, and the year of detection, a detailed SNP analysis using the CFSAN SNP Pipeline (https://peerj.com/articles/cs-20/) was performed for each ST with 5 or more isolates. For suspected duplicates (eg, twins or recurrence >28 days), only 1 isolate was analyzed. The SNP matrix obtained from the CFSAN SNP Pipeline was subsequently input into GraphSNP [30], which generated a minimum spanning tree and visualized a cluster based on a threshold of 15, as described in a previous study [8].

The details of the whole-genome sequencing analysis are described in the Supplementary Materials.

### Statistical Analysis

We used the Cochran–Armitage trend test to examine the temporal changes in the variables. The period before 2010 was excluded from the analysis because of the small number of isolates. *P* < .05 was considered significant. All analyses were performed using R version 4.4.3 (R Foundation for Statistical Computing, Vienna, Austria) [31].

### Ethical Considerations

The Institutional Ethics Committee of the National Institute of Infectious Diseases approved this study (Approval Number: 1512).

## RESULTS

### Patient Background

During the study period, 237 cases of pediatric invasive GBS infections were registered at 35 hospitals in 20 prefectures (Supplementary Figure 1). The characteristics of the patients, including the types of specimens analyzed, are shown in Table 1. No apparent differences were observed after 2011, when the number of samples collected became sufficient.

**Table 1.** Characteristics of Pediatric Patients With Invasive *Streptococcus Agalactiae* Infection in Japan, 2004–2023

Characteristic	No. of Isolates, N (%)				
	Total cases (N = 237)	Jan 2004 to Dec 2010 (N = 4)	Jan 2011 to Dec 2015 (N = 55)	Jan 2016 to Dec 2020 (N = 84)	Jan 2021 to May 2023 (N = 94)
Male	119 (50.2)	3 (75.0)	24 (43.6)	44 (52.4)	48 (51.1)
Age, day, median [IQR]	27.0 [13.0–55.0]	26.0 [1.5–61.3]	26.0 [14.5–49.5]	24.5 [14.0–46.8]	32.0 [13.0–63.0]
Age group					
0–6 d (EOD)	41 (17.3)	2 (50.0)	8 (14.5)	12 (14.3)	19 (20.2)
7–89 d (LOD)	163 (68.8)	1 (25.0)	40 (72.7)	61 (72.6)	61 (64.9)
>89 d (VLOD)	33 (13.9)	1 (25.0)	7 (12.7)	11 (13.1)	14 (14.9)
Types of specimens					
Blood	191 (80.6)	4 (100)	43 (78.2)	72 (85.7)	72 (76.6)
Cerebrospinal fluid	46 (19.4)	0 (0)	12 (21.8)	12 (14.3)	22 (23.4)

Abbreviations: EOD, early-onset disease; IQR, interquartile range; LOD, late-onset disease; VLOD, very late-onset disease.

### Serotype

Throughout the study period, serotype III was the most common (48.1%), followed by Ia (27.4%) and Ib (12.2%; Table 2). Between January 2011 and May 2023, the proportion of serotype VI decreased (*P* = .034), whereas that of serotype IV increased (*P* = .0089). Serotype IV first appeared in 2019 and peaked at 9.1% in 2022–2023 (Supplementary Figure 2). Serotype IV was detected in all age groups (Supplementary Figure 3).

**Table 2.** Trends in Serotype Distribution by Study period

Serotype	No. of Isolates, N (%)					*P* Value^a^
	Total cases (N = 237)	Jan 2004 to Dec 2010 (N = 4)	Jan 2011 to Dec 2015 (N = 55)	Jan 2016 to Dec 2020 (N = 84)	Jan 2021 to May 2023 (N = 94)	
Ia	65 (27.4)	1 (25.0)	14 (25.5)	18 (21.4)	32 (34.0)	.17
Ib	29 (12.2)	0 (0)	7 (12.7)	9 (10.7)	13 (13.8)	.77
II	2 (0.8)	0 (0)	0 (0)	2 (2.4)	0 (0)	.76
III	114 (48.1)	2 (50.0)	27 (49.1)	51 (60.7)	34 (36.2)	.049
IV	10 (4.2)	0 (0)	0 (0)	2 (2.4)	8 (8.5)	.0089
V	13 (5.5)	0 (0)	4 (7.3)	2 (2.4)	7 (7.4)	.76
VI	2 (0.8)	0 (0)	2 (3.6)	0 (0)	0 (0)	.034
VII	0 (0)	0 (0)	0 (0)	0 (0)	0 (0)	NA
VIII	2 (0.8)	1 (25.0)	1 (1.8)	0 (0)	0 (0)	.13
IX	0 (0)	0 (0)	0 (0)	0 (0)	0 (0)	NA

Abbreviation: NA, not applicable.

^a^Using Cochran–Armitage trend test except the periods between January 2004 and December 2010.

### Genotype

**Figure 1.**
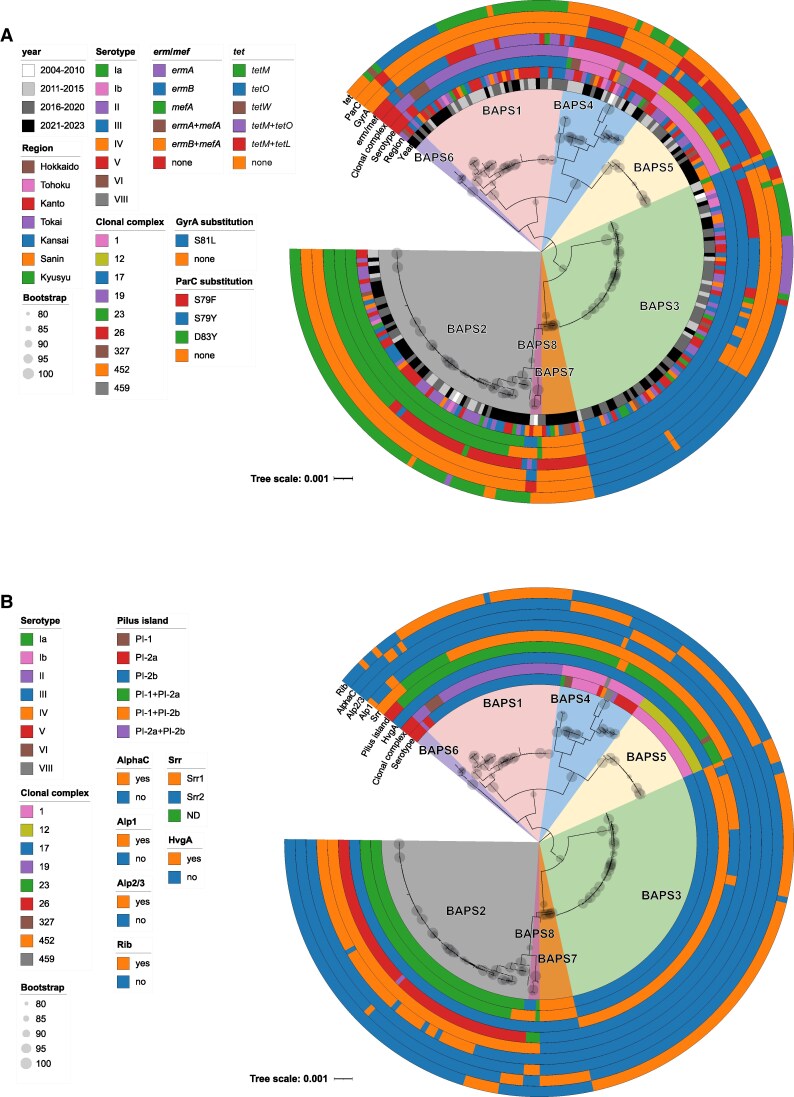
A maximum likelihood tree for all 237 isolates tested in this study. The clusters defined by BAPS are shown on the tree. *A*, A phylogenetic tree including metadata on resistance genes, year of detection, region, serotype, and genotype (clonal complex). *B*, A phylogenetic tree including metadata on major candidate GBS vaccine targets, serotypes, and genotypes (clonal complex).

The 237 isolates tested were assigned to 27 STs and divided into 8 BAPS clusters, with each cluster predominantly composed of isolates belonging to a single clonal complex (CCs) (Figure 1). CC17 (31.6%, BAPS3) was the most prevalent of the clusters and all isolates of CC17 were serotype III. CC1 (8.4%, BAPS4) was highly diverse in terms of serotype, comprising 6 serotypes (Ia, Ib, II, V, VI, and VIII). The highest whole genome diversity calculated by pairwise Jaccard distances was also observed in ST1 isolates (Supplementary Table 2). Of the 10 serotype IV isolates detected since 2019, 9 belonged to CC452 (6 ST452 and 3 single-locus variants of ST452), and 1 belonged to CC459 (ST196).

### Antimicrobial Susceptibility

All isolates were 100% susceptible to PEN and CTX (Table 3 and Supplementary Table 3). These isolates harbored either PBP2X types 1, 2, 4, or 5, or one of the 3 novel PBP2X types identified in this study (Supplementary Tables 3–5). The resistance rates to ERY, CLI, LVX, and TET were 61.2%, 43.5%, 30.0%, and 75.5%, respectively. No significant changes in resistance rates were observed during the study period (*P* = .23, .32, .06, and .50; Table 3). Among the CLI-resistant isolates, constitutive resistance (n = 96, 40.5%) was predominant compared to inducible resistance (n = 7, 3.0%). The predominant gene in both ERY- and constitutively CLI-resistant isolates was *ermB*, which was detected in 87 of 145 (60.0%) and 86 of 96 (89.6%) isolates, respectively. Notably, 59 of the 75 CC17 (BAPS3) isolates (78.7%) harbored *ermB* (Figure 1*A* and Supplementary Table 5). In contrast, 35 of 61 CC23 (BAPS2) isolates (57.4%) harbored only *mefA*/*msrD*. Sixty-five of the 71 LVX-resistant isolates (91.5%) had double substitutions in GyrA (S81L) and ParC (S79F/Y). All 22 CC12 (BAPS5) isolates had amino acid substitutions and exhibited resistance to levofloxacin (Figure 1*A* and Supplementary Table 5). Among the TET-resistant isolates, *tetO* was detected in 75 of 179 (41.9%), of which 60 (80.0%) were serotype III/CC17 isolates.

**Table 3.** Trends in Antibiotic Resistant Rate by Study Period

Antibiotic	No. of Resistant Isolates, N (%)					*P* value^a^
	Total cases (N = 237)	Jan 2004 to Dec 2010 (N = 4)	Jan 2011 to Dec 2015 (N = 55)	Jan 2016 to Dec 2020 (N = 84)	Jan 2021 to May 2023 (N = 94)	
Penicillin	0 (0)	0 (0)	0 (0)	0 (0)	0 (0)	NA
Cefotaxime	0 (0)	0 (0)	0 (0)	0 (0)	0 (0)	NA
Erythromycin	145 (61.2)	1 (25.0)	28 (50.9)	57 (67.9)	59 (62.8)	.23
Clindamycin	103 (43.5)	1 (25.0)	17 (30.9)	45 (53.6)	40 (42.6)	.32
Levofloxacin	71 (30.0)	1 (25.0)	10 (18.2)	28 (33.3)	32 (34.0)	.060
Tetracycline	179 (75.5)	3 (75.0)	41 (74.5)	68 (81.0)	67 (71.3)	.50

Abbreviation: NA, not applicable.

^a^Using Cochran–Armitage trend test except the periods between January 2004 and December 2010.

### Surface Protein Determinants

PI-1 + PI-2a was the most common pilus island (34.6%). Serotype III/CC17 isolates harbored either PI-2b alone (80.0%) or PI-1 + PI-2b (20.0%). The former was also detected in serotype IV/CC452 isolates (Figure 1*B* and Supplementary Table 6). Serine-rich repeat glycoprotein determinants (*srr1* or *srr2*) were detected in 226 of the 237 isolates (95.4%), with *srr1* being more predominant (n = 142, 59.9%) across various CCs. Almost all *srr2* were identified in serotype III/CC17 isolates, whereas 9 serotype IV/CC452 isolates also harbored *srr2* (Figure 1*B* and Supplementary Table 6). Although most isolates (94.9%) possessed at least one protein from the Alp family, the prevalence was lower in serotype III/ST529 and serotype V/ST26, at 75% and 25%, respectively. As for the other vaccine candidates, nearly all isolates harbored the screened proteins, except BibA, which was exclusively detected in serotype III/CC17 isolates (Supplementary Table 6).

### Vaccine Target Coverage Estimations

The distributions of the specific vaccine candidates are shown in Table 4. Among all isolates, 87.8% and 98.3% were covered by trivalent (serotypes Ia, Ib, and III) and hexavalent (Ia, Ib, II–V) polysaccharide vaccines, respectively. Hexavalent vaccine coverage increased significantly (*P* = .0093). The coverage rates of the pilus-based GBS-NN and GBS-NN/NN2 vaccines were 67.5% and 94.9%, respectively. There were no significant changes in the trend (*P* = .94 and.71).

**Table 4.** Trends in the Vaccine Coverage Rates by Study Period

Types of Vaccines	Coverage Rates, %					*P* Value^a^
	Total Cases (N = 237)	Jan 2004 to Dec 2010 (N = 4)	Jan 2011 to Dec 2015 (N = 55)	Jan 2016 to Dec 2020 (N = 84)	Jan 2021 to May 2023 (N = 94)	
Polysaccharide vaccines	…	…	…	…	…	…
Trivalent (Ia, Ib, and III)	87.8	75.0	87.3	92.9	84.0	.39
Hexavalent (Ia, Ib, II, III, IV, and V)	98.3	75.0	94.5	100	100	.0093
Pilus-based vaccines	…	…	…	…	…	…
GBS-NN vaccine^b^	67.5	25.0	63.6	73.8	66.0	.94
GBS-NN/NN2 vaccine^c^	94.9	100	94.5	96.4	93.6	.71

^a^Using Cochran–Armitage trend test except the periods between January 2004 and December 2010.

^b^Targeting N-terminal domains of the AlphaC and Rib.

^c^Targeting N-terminal domains of the Alp1, Alp2/3, AlphaC, and Rib.

### Temporal and Genetic Structure of Major GBS Lineages

We estimated the frequency of recombination events in STs with ≥10 isolates (ST1, 10, 17, 23, and 335) using Gubbins (Supplementary Figures 4 and Table 1). Three STs (ST10, 17, and 23) had average *r/m* ratios below 1. In contrast, ST1 showed the highest average *r/m* value of 4.024539. ST1 also exhibited the highest average *ρ*/*θ* value among the 5 STs.

In the Bayesian analysis, only STs with 20 or more isolates (ie, ST10, 17, and 23) were included to ensure sufficient analytical power. However, ST17 was excluded because no significant temporal signals were detected. The mutation rates of ST10 and ST23 isolates were estimated to be 1.26 × 10^−6^ (95% HPD interval 6.99 × 10^−7^ to 1.82 × 10^−6^) and 6.49 × 10^−7^ (95% HPD interval 4.73 to 8.20 × 10^−7^) substitutions per site per year, respectively. The divergence date of the root of ST10 and ST23 was estimated to be 1996.9 (95% HPD interval 1983.2 to 2004.4) and 1923.4 (95% HPD interval 1891.2 to 1947.3; Supplementary Figures 5 and 6).

### Potential Endemic Clusters and Horizontal Transmission

**Figure 2.**
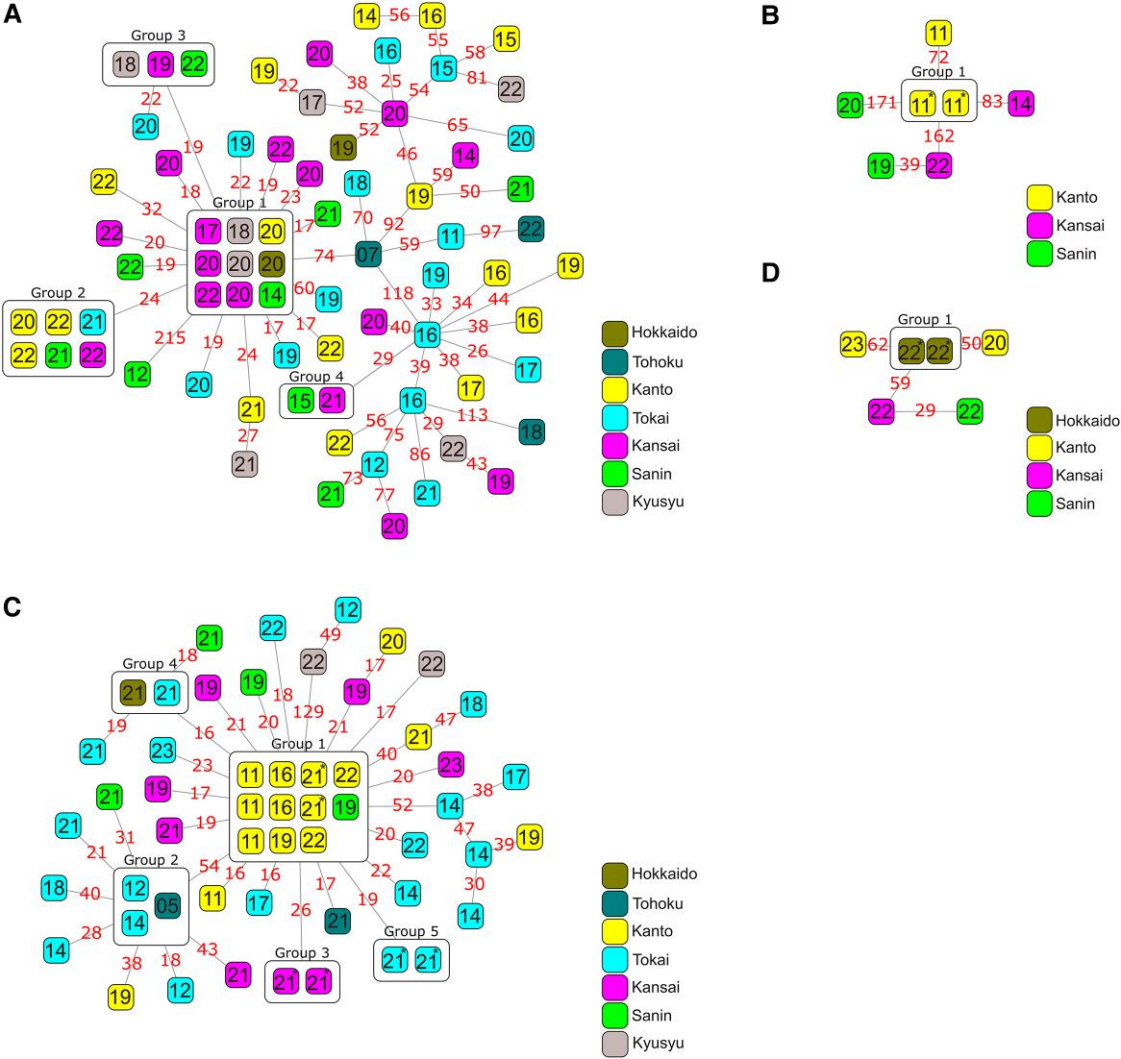
Minimum spanning trees created by GraphSNP with the metadata of detection regions, isolation year, and SNP distance. Each square represents a single isolate, with the number inside indicating the year of detection (eg, “22” denotes the year 2022), and the color of the square corresponds to the geographic region of the hospital from which the isolate was detected. For region names, see Supplementary Figure 1. Clustering (grouping) was performed using a threshold of 15 single nucleotide polymorphisms (SNPs). Isolates identified as belonging to the same cluster were enclosed within boxes in each phylogenetic tree, and a group number was assigned to each cluster. An asterisk indicates sets of isolates with 0–2 SNP differences, suggesting possible nosocomial horizontal transmission. The red numbers above the lines connecting the isolates indicate the number of SNPs between the respective strains, as calculated using the CFSAN SNP pipeline. *A*, ST17 (*B*) ST19 (*C*) ST23 (*D*) ST452.

We calculated pairwise SNP distances between isolates of ST1 (n = 12), 3 (n = 8), 10 (n = 19), 17 (n = 72), 19 (n = 7), 23 (n = 53), 27 (n = 7), 144 (n = 5), 335 (n = 17), and 452 (n = 6). Median pairwise SNP distances ranged from 37 (ST27) to 308 (ST1) (Supplementary Table 7). Violin plots suggested that the distribution of SNP counts was bimodal in several STs (Supplementary Figure 7). ST17 and ST23 had sufficient isolates for analysis and appeared to have a peak at the lower end, with SNP counts below 50. Therefore, we visualized the relationship between SNP counts among isolates, geographic regions, and years of isolation using GraphSNP and maximum likelihood trees (Figure 2, Supplementary Figures 8 and 9). We identified 12 endemic or potential horizontal transmission clusters across 4 STs (ST17, ST19, ST23, and ST452). The largest cluster consisted of 11 isolates. Some of the isolates within these clusters were separated by >10 years and were clearly recovered from distinct geographic regions. Among the 12 clusters, we found 5 groups of isolates (ie, 10 isolates) detected in the same hospitals within a 2-month period. All SNP differences between the set of complete genomes were minimal (0–4), although there was a 25.2 kb indel between 2 of the isolates (Supplementary Figure 10).

## DISCUSSION

Our nationwide retrospective GBS genomic surveillance study revealed that the hexavalent polysaccharide and protein-based GBS-NN/NN2 vaccines would provide comprehensive coverage against isolates from pediatric invasive GBS patients in Japan. Their coverage rate meets the WHO target for maternal GBS vaccines, which aims to cover at least 90% of current invasive disease isolates [11]. However, the coverage rate of the trivalent polysaccharide vaccine was 87.8%. This contrasts with questionnaire-based studies in Japan (2011–2020), which reported >90% coverage for invasive infant GBS [32]. The discrepancy in these results could be caused by the increase in the proportion of serotype IV isolates since 2019. Other countries have also reported increased invasive GBS due to serotype IV [33, 34]. In this study, 90% of the serotype IV isolates detected belonged to CC452, whereas serotype IV isolates reported in other countries were mainly classified as CC17, CC452, or CC459 [33–35]. An increase in serotype IV/CC452 has been reported since 2000 in the U.S. and Canada, suggesting that the isolates from Japan may be genetically related to those originating in North America [33, 34].

Several countries have reported clinical isolation of non-vaccine serotype strains. In central Taiwan, serotype VI/ST1 has been predominantly detected in infants with EOD and in colonizing pregnant women [36]. In Canada, invasive GBS infections in non-pregnant adults caused by serotype VIII/CC1 have increased since 2017 [37]. Although previous reports have shown that these serotypes are predominantly isolated from pregnant women in Japan [38], in the present study, we detected only 2 isolates each of serotypes VI and VIII. Several non-vaccine serotype strains mentioned above belong to the CC1. In our analysis, ST1 showed a markedly higher frequency of recombination events than did the other 4 major STs (ST10, ST17, ST23, and ST335), which could contribute to the emergence of novel serotype strains and resistant clones within CC1 [39]. In fact, ST1 showed a higher Jaccard distance than did the other STs, indicating greater diversity in gene content.

Resistance to macrolides and lincosamides, which are used as second-line treatments, is also a global issue [10]. In the U.S., where CLI-resistant GBS was noted as a concerning threat in 2019, resistance rates to ERY and CLI were approximately 60% and 40%, respectively [40]. Our study showed similar resistance rates, with no significant changes since 2011. Resistance to these antibiotics in GBS is primarily associated with the acquisition of methyltransferases encoded by *erm* genes or efflux pumps encoded by *mefA/msrD* [41]. Among the 237 isolates in our study, *ermB* was the most frequently detected, being identified in 87 isolates (36.7%) across 7 CCs. Recently, a multidrug-resistant CC17 GBS subclone has been detected in several countries [42–44]. This clone was found to lack PI-1 due to replacement by integrative and conjugative elements carrying resistance determinants, including *ermB* and *tetO*, and retained only PI-2b; the clone was characterized by resistance to ERY, CLI, and TET [42–44]. In the present study, among the 75 CC17 isolates, 59 (78.7%) exhibited similar genomic features. These findings raise concerns about the global spread of multidrug-resistant CC17 GBS clones and the implications for IAP in pregnant women with severe penicillin allergy.

Through SNP analysis and phylogenetic analysis, we identified 5 cases of nosocomial horizontal transmission, as well as clusters that have spread across wide geographic areas for extended periods. Due to protocol limits, patient details were unavailable; however, birth dates and hospital data strongly suggested nosocomial horizontal transmission. Although several studies have reported GBS outbreaks/transmission in hospitals, particularly in NICUs [45, 46], no previous studies have detected outbreaks/transmission based on a large-scale dataset, such as that used in this study. In our dataset, no cases of outbreaks/transmission were identified among the most prevalent genotype, ST17, whereas 3 of 5 outbreaks/transmission cases involved ST23 strains. This suggests that the propensity for transmission does not necessarily correlate with the invasiveness of the strain in neonates. In addition, we detected the potential circulation and spread of strains across regions and years in ST17 and ST23 using SNP analysis. In the absence of clear epidemiological links among patients from whom clustered strains were isolated, environmental sources such as food, water, and sewage systems may have contributed to transmission. ST283 GBS is widely recognized as a clone responsible for invasive GBS infections in humans, with freshwater fish identified as its primary source [8]. As a maximum of 14 SNPs was observed among GBS ST283 isolates from humans during the 2015 outbreak in Singapore, a threshold of 15 SNPs was used for clustering in this study. Although robust evidence is still lacking to define clear thresholds for genetic relatedness between GBS isolates, previous studies investigating the transmission and dissemination of *Enterobacterales* from a One Health perspective have proposed higher SNP thresholds ranging from 20 to 100 SNPs [47]. The evolutionary analyses we conducted yielded rates of 1.26 × 10^−6^ (ST10) and 6.49 × 10^−7^ (ST23) substitutions per site per year, which were considered reasonable compared to the previously reported rate of 2.7 × 10^−6^ for ST283 GBS [8]. Although data on the mutation rate of GBS was limited, our study provides stronger evidence that the mutation rate of GBS does not differ substantially from that of other major clinical pathogens such as *Staphylococcus aureus* (1.29 × 10^−6^ substitutions per site per year) [48], *Streptococcus pneumoniae* (1.57 × 10^−6^) [49], and *Escherichia coli* (1.00 × 10^−6^) [50]. We calculated the mutation rates for only 2 clones, and the average mutation rate across the entire GBS remains unknown. However, these findings may serve as a reference for defining thresholds for future outbreak detection efforts.

Our study collected bacterial isolates from institutions that had preserved isolates obtained from pediatric patients previously diagnosed with invasive GBS infections. Consequently, isolate preservation policies may vary among hospitals; for example, some may store only isolates from severe cases based on the attending physician's discretion. This variation may introduce bias in the data related to serotypes and antimicrobial susceptibility. Furthermore, the capacity of a hospital to preserve bacterial isolates may lead to sampling bias.

## CONCLUSION

This study demonstrated that the hexavalent polysaccharide and GBS-NN/NN2 vaccines would provide approximately 95% coverage for pediatric invasive GBS infections in Japan. Some GBS clones may circulate more broadly, and understanding their potential transmission pathways could help inform control measures and contribute to reducing invasive GBS infections in children.

## Supplementary Material

jiaf491_Supplementary_Data

## Supplementary Data

Supplementary materials are available at *The Journal of Infectious Diseases* online (http://jid.oxfordjournals.org/). Supplementary materials consist of data provided by the author that are published to benefit the reader. The posted materials are not copyedited. The contents of all supplementary data are the sole responsibility of the authors. Questions or messages regarding errors should be addressed to the author.
