# Supplementary material for: Prevalence of Candidate Vaccine Targets and Genomic Features of Pediatric Invasive Streptococcus Agalactiae in Japan
Source: J Infect Dis. 2025 Oct 9;233(1):e11–21. doi: 10.1093/infdis/jiaf491 (PMC12811887; doi:10.1093/infdis/jiaf491)
Supplement: jiaf491_Supplementary_Data [file jiaf491_supplementary_data.zip › Supplementary_materials.pdf]

Supplementary materials

Title: Prevalence of candidate vaccine targets and genomic features of pediatric invasive *Streptococcus agalactiae* in Japan.

Supplementary methods

Whole-genome sequencing

Genomic DNA was extracted using the QIAamp® DNA Mini Kit (QIAGEN, Hilden, Germany) according to the manufacturer's instructions. Multiplexed samples were sequenced on an Illumina NovaSeq X Plus platform for 300 cycles, generating 150-bp paired-end reads.

To obtain complete genomes, we extracted high molecular weight DNA with QIAGEN Genomic-tip and performed Nanopore long-read sequencing using R10.4 flow cell.

Bioinformatics

Basic in-silico analysis

After the acquisition of raw reads from illumina NovaSeqX Plus platform for 300 clycles, we first removed index and adapter sequences with low quality reads (<Q25) using fastp [1]. Then, we assembled the reads using shovill v1.0.9 (https://github.com/tseemann/shovill) with --minlen 300 option. We used these contigs for MLST (mlst [https://github.com/tseemann/mlst]), resistance gene detection using abricate v1.0.1 with NCBI database (database download was conducted on October 21th, 2024), and surface protein (hypervirulent GBS adhesin [HvgA], Srr proteins [Srr1 and Srr2], members of Alp family [AlphaC, Alp1, Alp2/3, and Rib], pilus islands [PI-1, PI-2A, and PI-2B]) detection following the method described previously by Metcalf et.al [2]. In addition, we screened for other candidate vaccine targets suggested by Gent et al [3]. To detect the targets, we used Blast+ v 2.14 with ≥90% identity and coverage parameters with reference protein sequences described below.

We followed clonal complex (CC) definition published on PubMLST website (https://pubmlst.org/).

Long reads were filtered using Nanofilt v2.8.0 [4] with parameters of Q≥10 and minimum read length of 1,000 bps. We conducted hybrid assembly using flye v2.9.0 [5] with standard parameters.

Pan-genome analysis and Phylogenetic tree reconstruction

In phylogenetic analysis, we first reconstructed a maximum likelihood (ML) tree using RAxML Next Generation v1.1 [6]. The core genome of 237 GBS isolates was identified using Prokka 1.14.6 [7] and panaroo v1.3.4 [8] with the strict mode. The optimal nucleotide substitution model for maximum likelihood analysis was determined using ModelTest-NG v0.1.7 [9]. Similarly, we identified core-genome of each ST with more than 10 isolates to calculate pairwise Jaccard distances.

To construct the sub-clusters of the ML tree, we performed BAPS analysis using rhierBAPS v1.0.1 [10] with mex.depth = 2 and n.pops = 40. The input file of rhierBAPS was generated by panaroo v1.3.4 [8] with a strict mode.

Afterward, we reconstructed maximum-likelihood trees of each ST with 20 of more isolates using Gubbins v3.4 [11] with standard parameters. In the preparation of the input files for Gubbins analysis, we used Snippy v4.6.0 (https://github.com/tseemann/snippy) and the reference sequences are listed in Table S1. To avoid mapping bias, we selected a reference genome from a strain belonging to the same sequence type that was registered as a RefSeq in the NCBI database and originated from a country other than Japan.

Bayesian analysis

Since ST10, ST17, and ST23 each comprised more than 20 isolates in the collection, we decided to estimate the mutation rate of each clone and perform root dating accordingly. For each clone, sequencing reads were mapped to the corresponding reference sequences listed in Table S1 using Snippy. Recombinant regions were identified using Gubbins and subsequently masked using Bedtools v2.30.0 [12]. Additionally, regions corresponding to mobile genetic elements, identified with geNomad v1.8.1 [13], were also masked. These recombination-censored multi-FASTA files were used as input to generate BEAST2 input files using BEAUti. First, we assessed whether each input file contained a significant temporal signal by performing a permutation test implemented in BactDating v1.1 [14]. As shown in Figure S5, no significant temporal signal was detected for ST17; therefore, subsequent analyses were conducted only for ST10 and ST23. The model was selected through comparisons of the marginal likelihood using path sampling and stepping stone-based marginal likelihood estimation for a strict clock and an uncorrelated relaxed clock in a molecular clock model and a constant population size structured coalescent model and Bayesian skyline plot in a tree prior model. Consequently, we selected an uncorrelated relaxed clock and a Bayesian skyline plot model for the ST10 and an uncorrelated relaxed clock and a constant population size structured coalescent model for the ST23. To obtain an effective sample size (ESS) greater than 200 for all factors, we set the MCMC lengths for ST10 and ST23 to 1.0×10^8 and 2.0×10^8, respectively. We specified a general-time-reversible substitution model with site rate heterogeneity modeled across four gamma distributions (GTR+Γ4) for all analyses.

SNP analysis

To calculate the pairwise SNP distances within each ST, we analyzed trimmed reads using the CF-SAN SNP Pipeline (CFSAN SNP Pipeline: an automated method for constructing SNP matrices from next-generation sequence data). The analysis was performed for STs that included two or more isolates, resulting in a total of 14 STs. In this analysis, when two isolates were recovered either from twins or from the same patient more than 28 days apart, and shared the same serotype and ST, only one of the isolates was included in the analysis. For STs containing four or fewer isolates, SNP distance matrices were manually inspected, and no clusters—defined as groups of isolates with genetic links of 15 or fewer SNPs—were identified. For STs with five or more isolates, the SNP distance matrices were used as input for GraphSNP [15]. By integrating this data with information on the geographic locations and years of isolation, we visualized the presence of clusters within each ST, as well as the transmission patterns of individual strains across regions and over time. The threshold for defining clusters in GraphSNP was set to 15 SNPs, based on the previous study of GBS-ST283 outbreak in Singapore in 2015 [16].

As a result of the analysis, we identified several sets of isolates with pairwise SNP counts ranging from 0 to 3. For these isolates, we closed the genomes using the aforementioned method and conducted a more detailed SNP analysis using MUMmer v4.0.1 [17]. The visualization of the sequences was performed using LoVis4u v0.0.11 [18].

Reference

1. Chen S, Zhou Y, Chen Y, Gu J. fastp: an ultra-fast all-in-one FASTQ preprocessor. Bioinformatics **2018**; 34:i884-90.

2. Metcalf BJ, Chochua S, Gertz RE, Jr., et al. Short-read whole genome sequencing for determination of antimicrobial resistance mechanisms and capsular serotypes of current invasive Streptococcus agalactiae recovered in the USA. Clin Microbiol Infect **2017**;

23:574. e7-14.
3. Gent V, Lu YJ, Lukhele S, et al. Surface protein distribution in Group B Streptococcus isolates from South Africa and identifying vaccine targets through in silico analysis. Sci Rep **2024**; 14:22665.
4. De Coster W, D'Hert S, Schultz DT, Cruts M, Van Broeckhoven C. NanoPack: visualizing and processing long-read sequencing data. Bioinformatics **2018**; 34:2666-9.
5. Kolmogorov M, Yuan J, Lin Y, Pevzner PA. Assembly of long, error-prone reads using repeat graphs. Nat Biotechnol **2019**; 37:540-6.
6. Kozlov AM, Darriba D, Flouri T, Morel B, Stamatakis A. RAxML-NG: a fast, scalable and user-friendly tool for maximum likelihood phylogenetic inference. Bioinformatics **2019**; 35:4453-5.
7. Seemann T. Prokka: rapid prokaryotic genome annotation. Bioinformatics **2014**; 30:2068-9.
8. Tonkin-Hill G, MacAlasdair N, Ruis C, et al. Producing polished prokaryotic pangenomes with the Panaroo pipeline. Genome Biol **2020**; 21:180.
9. Darriba D, Posada D, Kozlov AM, Stamatakis A, Morel B, Flouri T. ModelTest-NG: A New and Scalable Tool for the Selection of DNA and Protein Evolutionary Models. Mol Biol Evol **2020**; 37:291-4.
10. Tonkin-Hill G, Lees JA, Bentley SD, Frost SDW, Corander J. RhierBAPS: An R implementation of the population clustering algorithm hierBAPS. Wellcome Open Res **2018**; 3:93.
11. Croucher NJ, Page AJ, Connor TR, et al. Rapid phylogenetic analysis of large samples of recombinant bacterial whole genome sequences using Gubbins. Nucleic Acids Res **2015**; 43:e15.
12. Quinlan AR, Hall IM. BEDTools: a flexible suite of utilities for comparing genomic features. Bioinformatics **2010**; 26:841-2.
13. Camargo AP, Roux S, Schulz F, et al. Identification of mobile genetic elements with geNomad. Nat Biotechnol **2024**; 42:1303-
14. Didelot X, Croucher NJ, Bentley SD, Harris SR, Wilson DJ. Bayesian inference of ancestral dates on bacterial phylogenetic trees. Nucleic Acids Res **2018**; 46:e134.
15. Permana B, Beatson SA, Forde BM. GraphSNP: an interactive distance viewer for investigating outbreaks and transmission networks using a graph approach. BMC Bioinformatics **2023**; 24:209.
16. Kalimuddin S, Chen SL, Lim CTK, et al. 2015 Epidemic of Severe Streptococcus agalactiae Sequence Type 283 Infections in Singapore Associated With the Consumption of Raw Freshwater Fish: A Detailed Analysis of Clinical, Epidemiological, and Bacterial Sequencing Data. Clin Infect Dis **2017**; 64:S145-52.
17. Marçais G, Delcher AL, Phillippy AM, Coston R, Salzberg SL, Zimin A. MUMmer4: A fast and versatile genome alignment system. PLoS Comput Biol **2018**; 14:e1005944.
18. Egorov AA, Atkinson GC. LoVis4u: a locus visualization tool for comparative genomics and coverage profiles. NAR Genom Bioinform **2025**; 7:lqaf009.

Supplementary figures

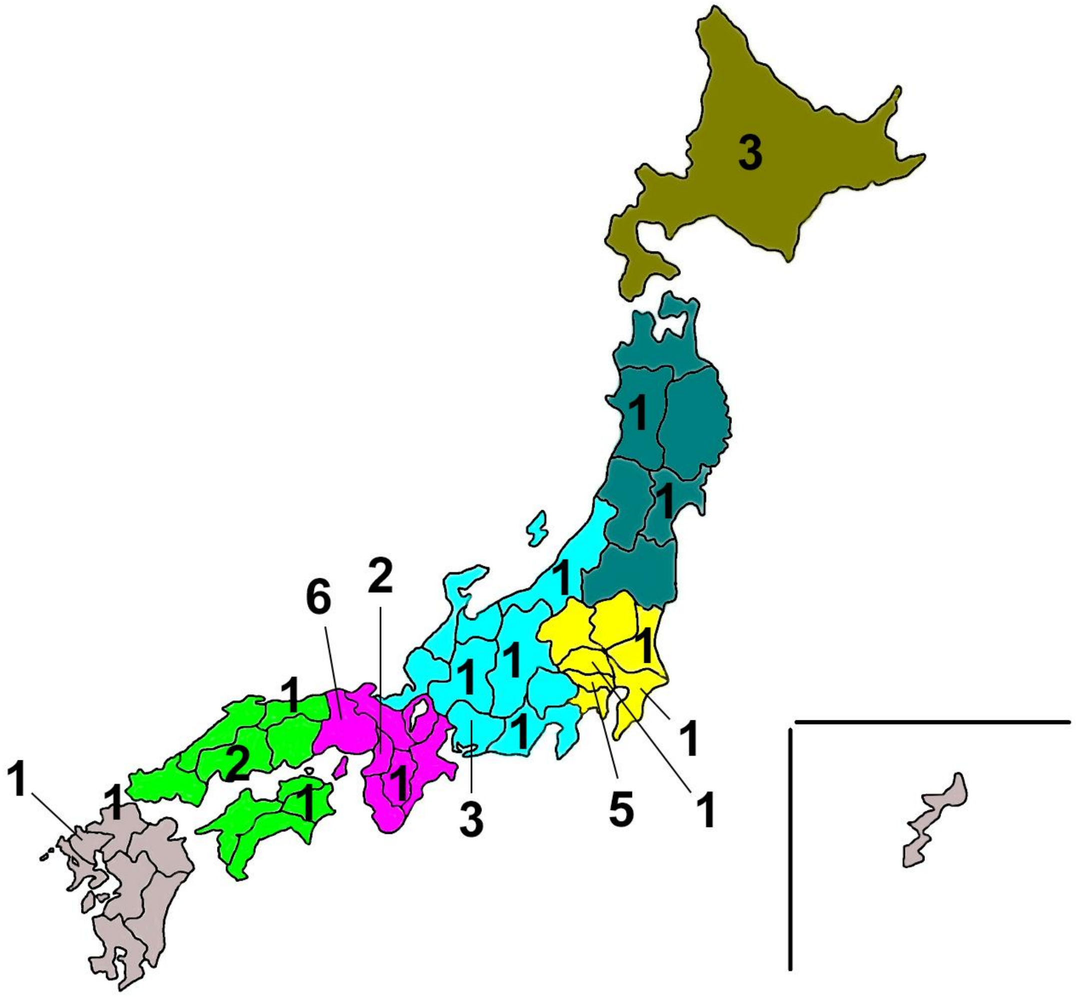

Figure S1. Geographical distribution of 35 hospitals participating the present retrospective pediatric invasive *Streptococcus agalactiae* infection surveillance study for in Japan. The hospitals were widely distributed across 20 prefectures in various regions of Japan. The map is highlighted according to geographic regions; Hokkaido, dark olive green; Tohoku, dark cyan; Tokai, light blue; Kanto, yellow; Kansai, pink; Sanin, green; Kyusyu, light brown.

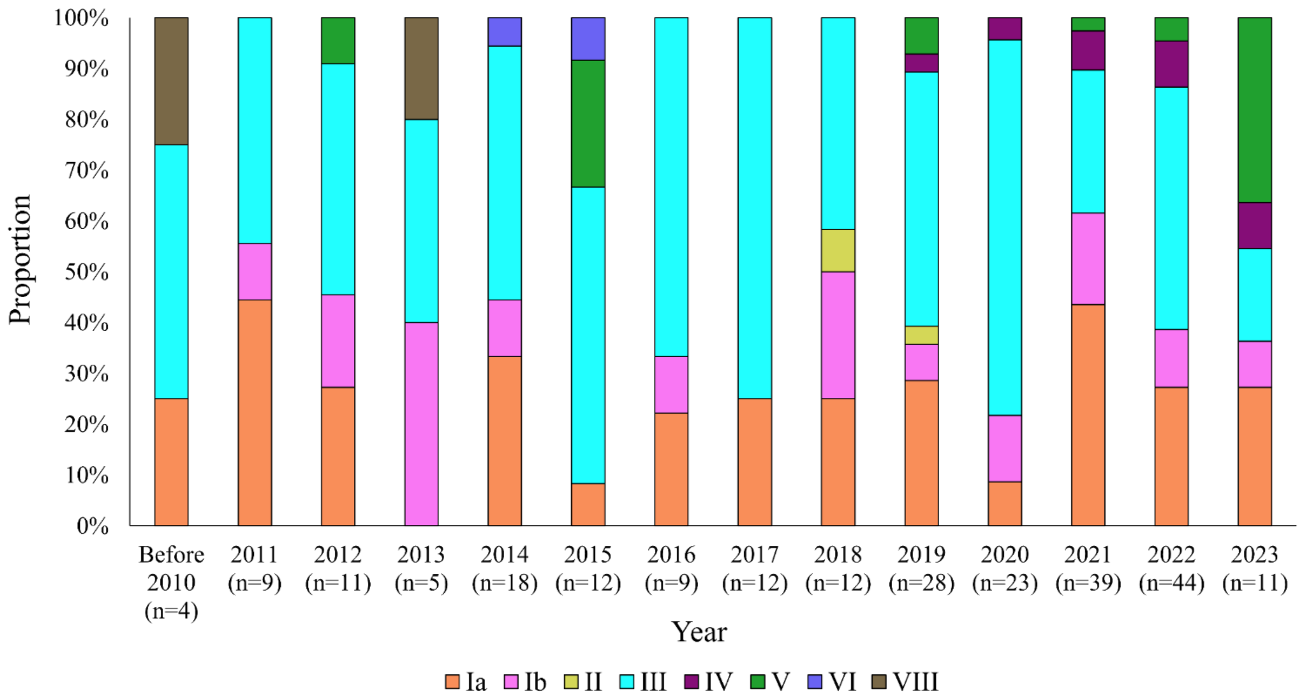

Figure S2. Annual distribution of serotypes of 237 *Streptococcus agalactiae* isolates, 2004-2023. In most years, serotype III was predominant; however, in 2021, serotype Ia was the most common, with 17 isolates. In 2023, serotypes V was the most common, with four isolates. Serotype IV was first identified in 2019 and reached its highest prevalence in 2022 and 2023. Serotypes VII and IX were not detected throughout the study period.

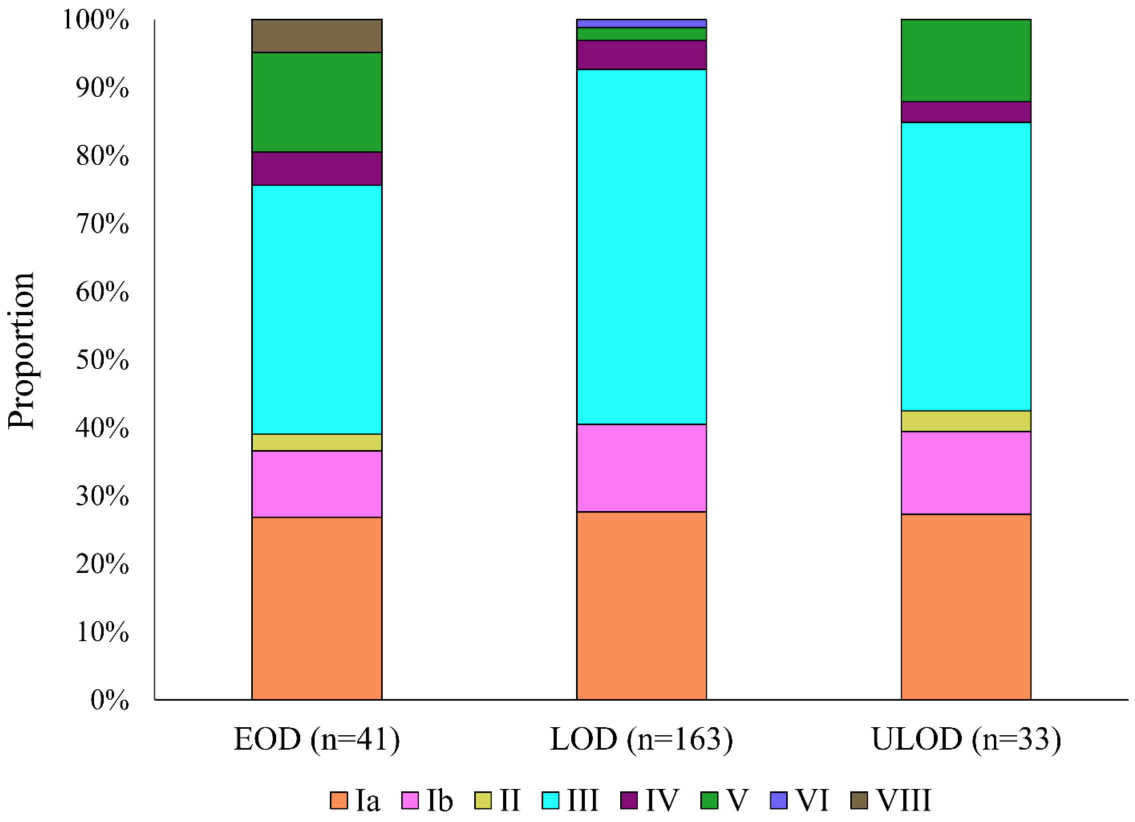

Figure S3. Distribution of 237 *Streptococcus agalactiae* serotypes by age group. Among all age groups, serotype III had the highest prevalence. Serotype IV was detected in isolates from all age groups. Serotype VI was detected only in LOD cases, whereas serotype VIII was detected only in EOD cases.

EOD, early onset disease; LOD, late onset disease; ULOD, ultra late onset disease

(a)

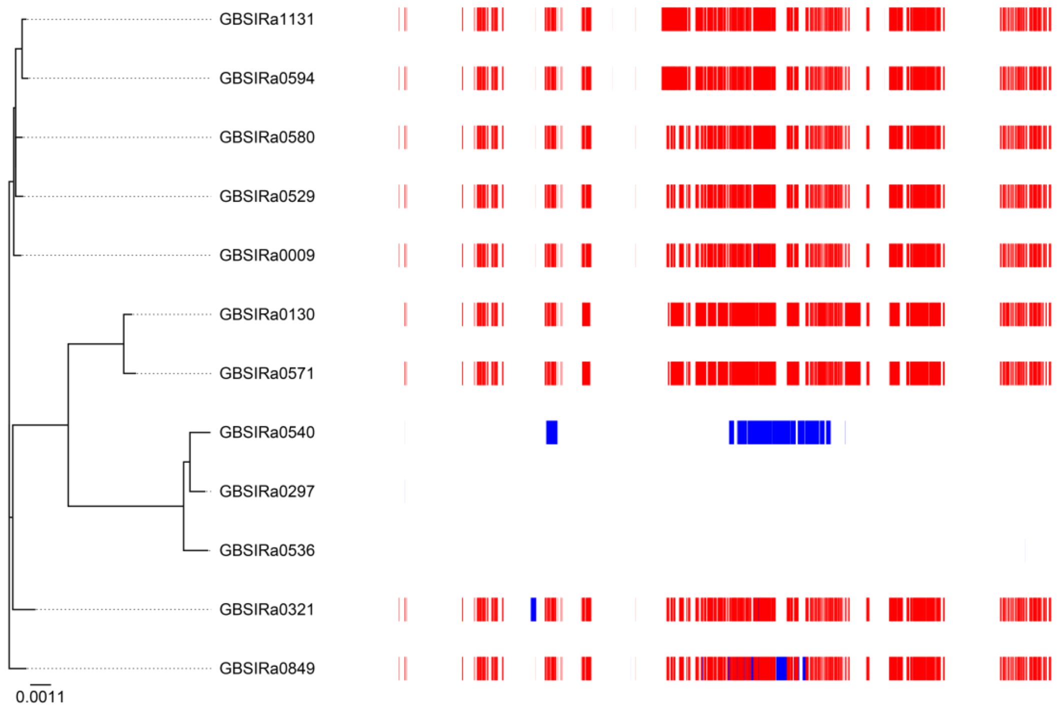
GBSIRa1131
GBSIRa0594
GBSIRa0580
GBSIRa0529
GBSIRa0009
GBSIRa0130
GBSIRa0571
GBSIRa0540
GBSIRa0297
GBSIRa0536
GBSIRa0321
GBSIRa0849
0.0011

(b)

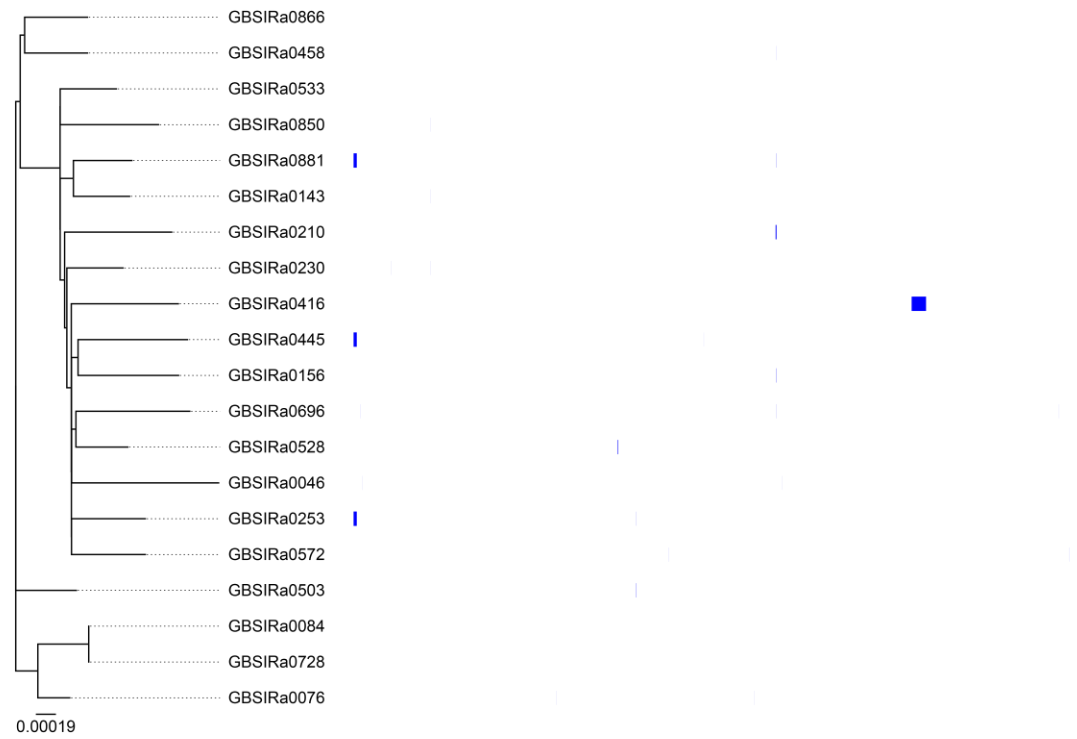
GBSIRa0866
GBSIRa0458
GBSIRa0533
GBSIRa0850
GBSIRa0881
GBSIRa0143
GBSIRa0210
GBSIRa0230
GBSIRa0416
GBSIRa0445
GBSIRa0156
GBSIRa0696
GBSIRa0528
GBSIRa0046
GBSIRa0253
GBSIRa0572
GBSIRa0503
GBSIRa0084
GBSIRa0728
GBSIRa0076
0.00019

(c)

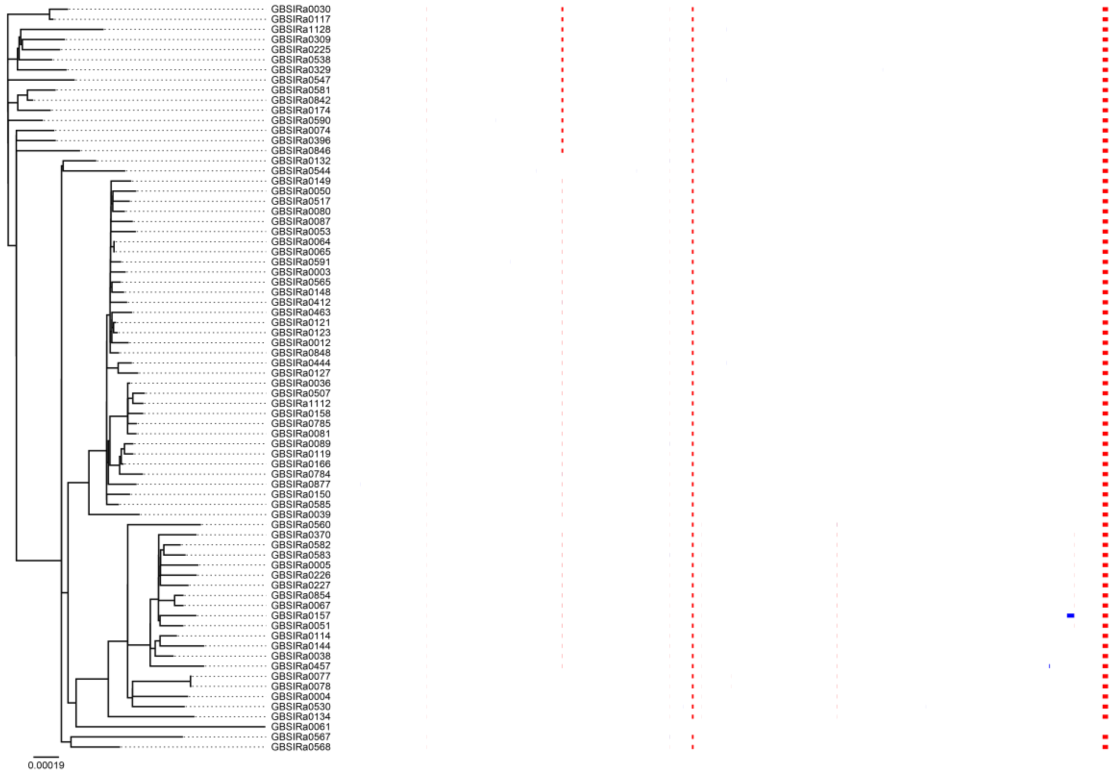
GBSIRa0030
GBSIRa0117
GBSIRa1128
GBSIRa0309
GBSIRa0225
GBSIRa0538
GBSIRa0329
GBSIRa0547
GBSIRa0581
GBSIRa0842
GBSIRa0174
GBSIRa0590
GBSIRa0074
GBSIRa0396
GBSIRa0846
GBSIRa0132
GBSIRa0544
GBSIRa0149
GBSIRa0050
GBSIRa0517
GBSIRa0080
GBSIRa0087
GBSIRa0053
GBSIRa0064
GBSIRa0065
GBSIRa0591
GBSIRa0003
GBSIRa0565
GBSIRa0148
GBSIRa0412
GBSIRa0463
GBSIRa0121
GBSIRa0123
GBSIRa0012
GBSIRa0848
GBSIRa0444
GBSIRa0127
GBSIRa0036
GBSIRa0507
GBSIRa1112
GBSIRa0158
GBSIRa0785
GBSIRa0081
GBSIRa0089
GBSIRa0119
GBSIRa0166
GBSIRa0784
GBSIRa0877
GBSIRa0150
GBSIRa0585
GBSIRa0039
GBSIRa0560
GBSIRa0370
GBSIRa0582
GBSIRa0583
GBSIRa0005
GBSIRa0226
GBSIRa0227
GBSIRa0854
GBSIRa0067
GBSIRa0157
GBSIRa0051
GBSIRa0114
GBSIRa0144
GBSIRa0038
GBSIRa0457
GBSIRa0077
GBSIRa0078
GBSIRa0004
GBSIRa0530
GBSIRa0134
GBSIRa0061
GBSIRa0567
GBSIRa0568
0.00019

(d)

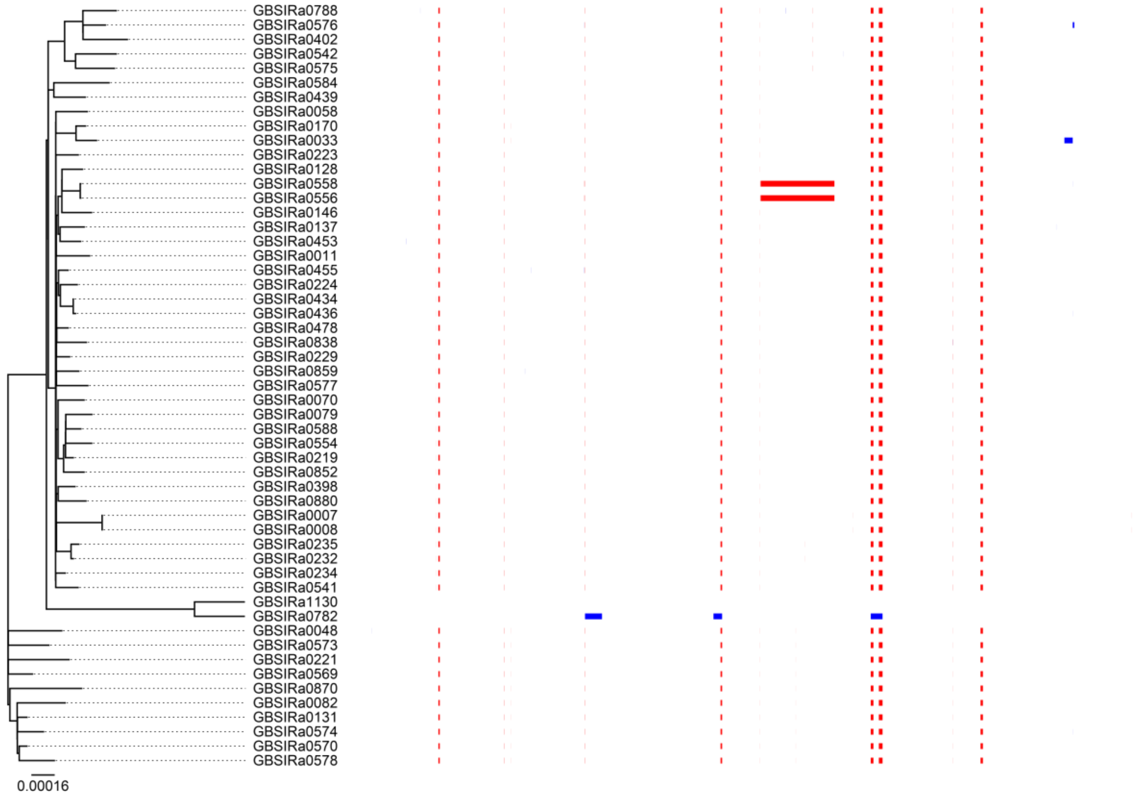
GBSIRa0788
GBSIRa0576
GBSIRa0402
GBSIRa0542
GBSIRa0575
GBSIRa0584
GBSIRa0439
GBSIRa0058
GBSIRa0170
GBSIRa0033
GBSIRa0223
GBSIRa0128
GBSIRa0558
GBSIRa0556
GBSIRa0146
GBSIRa0137
GBSIRa0453
GBSIRa0011
GBSIRa0455
GBSIRa0224
GBSIRa0434
GBSIRa0436
GBSIRa0478
GBSIRa0838
GBSIRa0229
GBSIRa0859
GBSIRa0577
GBSIRa0070
GBSIRa0079
GBSIRa0588
GBSIRa0554
GBSIRa0219
GBSIRa0852
GBSIRa0398
GBSIRa0880
GBSIRa0007
GBSIRa0008
GBSIRa0235
GBSIRa0232
GBSIRa0234
GBSIRa0541
GBSIRa1130
GBSIRa0782
GBSIRa0048
GBSIRa0573
GBSIRa0221
GBSIRa0569
GBSIRa0870
GBSIRa0082
GBSIRa0131
GBSIRa0574
GBSIRa0570
GBSIRa0578
0.00016

(e)

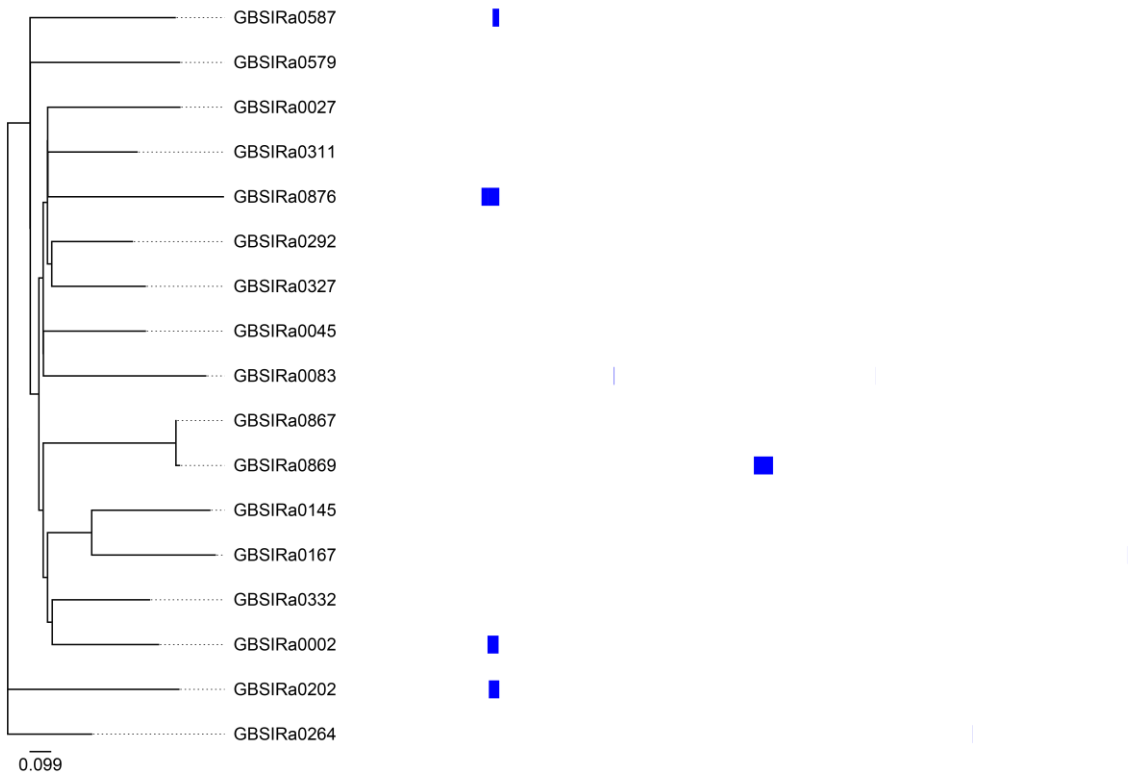

Figure S4. A maximum likelihood tree reconstructed by Gubbins. In each phylogenetic tree, recombination regions are displayed on the right-hand side with blue and red blocks. Blue blocks are unique to a single isolate while red blocks are shared by multiple isolates. (a) ST1 (b) ST10 (c) ST17 (d) ST23 (e) ST335.

(a)

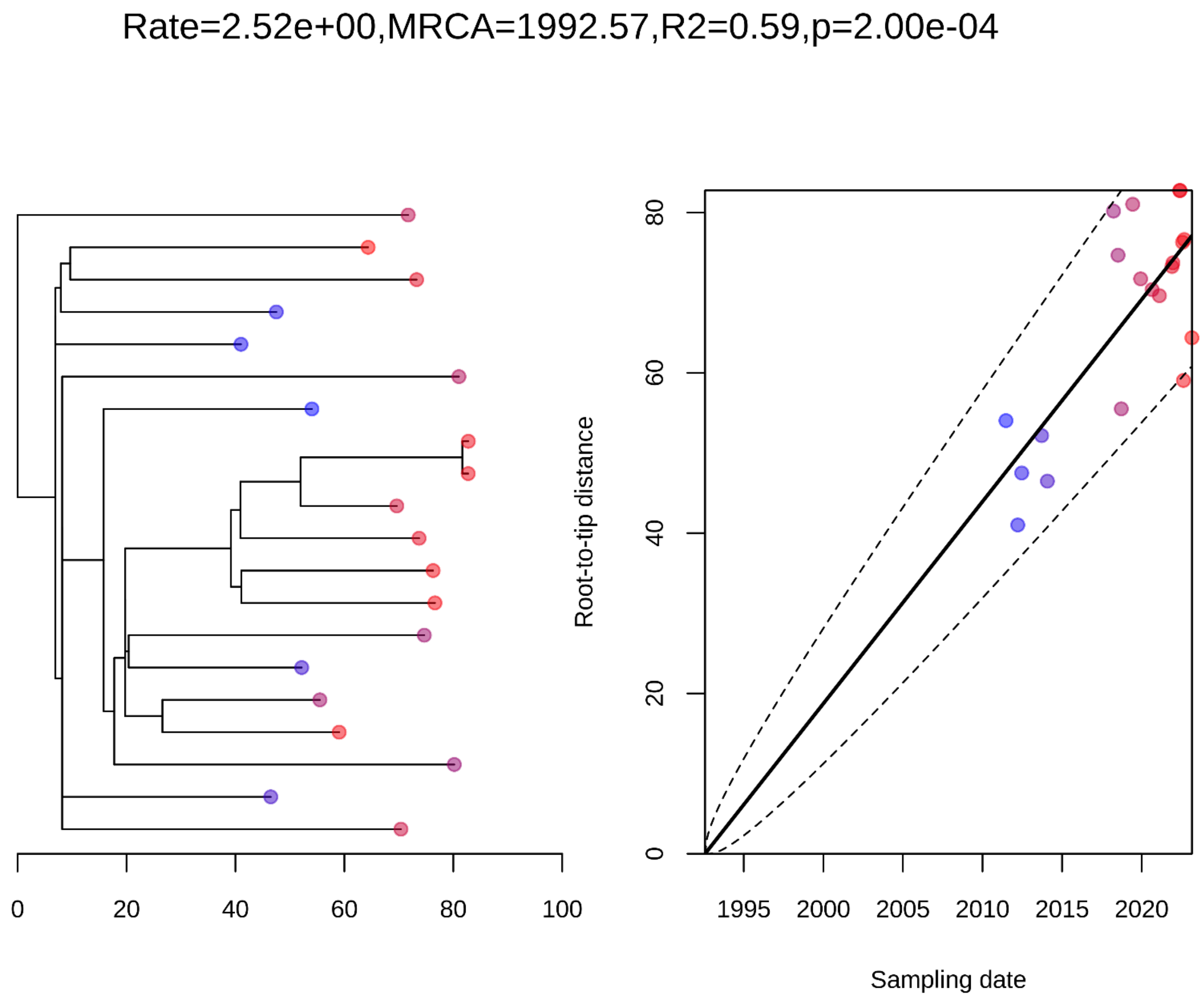
Rate=2.52e+00,MRCA=1992.57,R2=0.59,p=2.00e-04
0
20
40
60
80
100
Root-to-tip distance
0
20
40
60
80
1995
2000
2005
2010
2015
2020
Sampling date

(b)

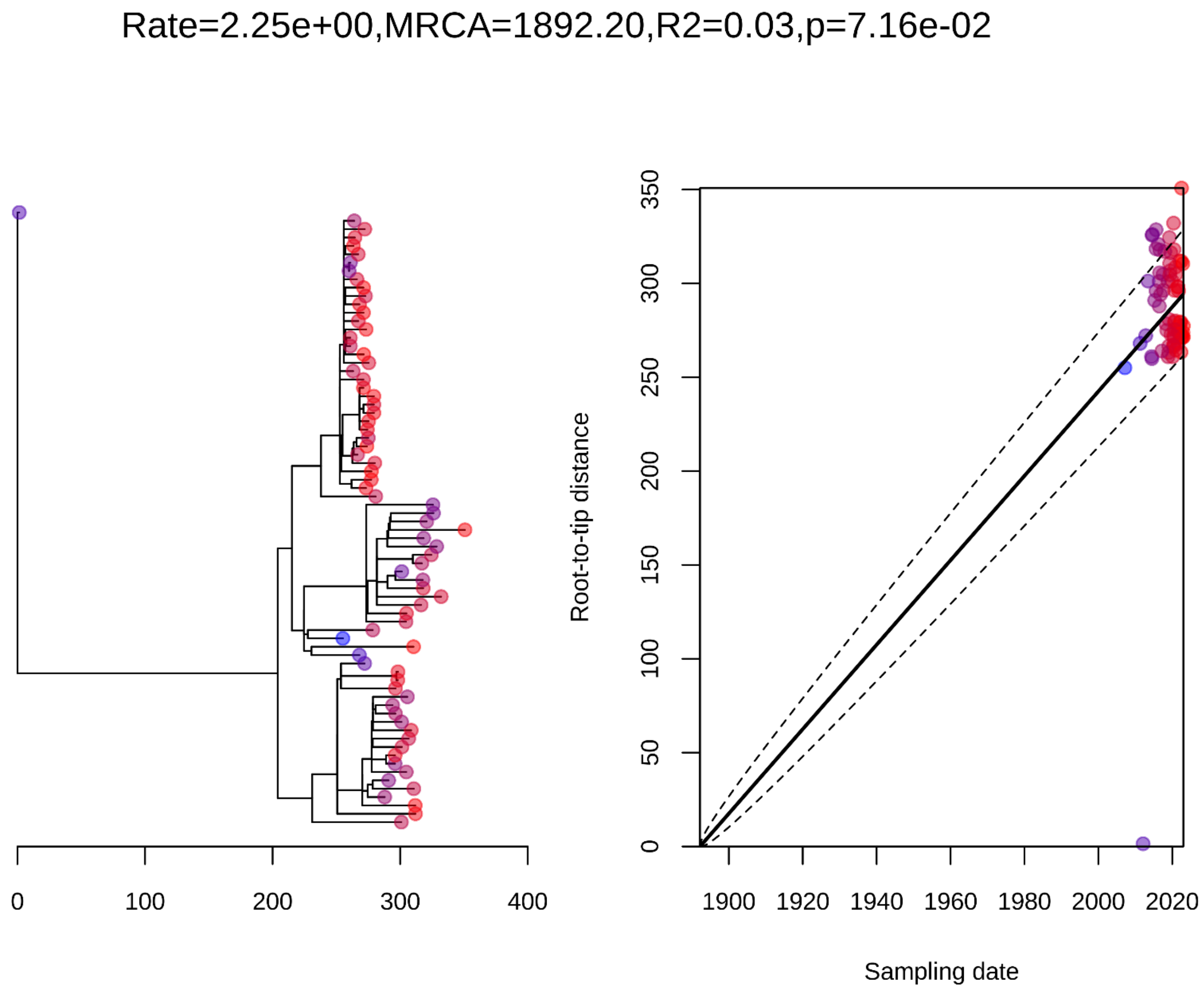

Rate=2.25e+00,MRCA=1892.20,R2=0.03,p=7.16e-02
0
100
200
300
400
Root-to-tip distance
0
50
100
150
200
250
300
350
1900
1920
1940
1960
1980
2000
2020
Sampling date

(c)

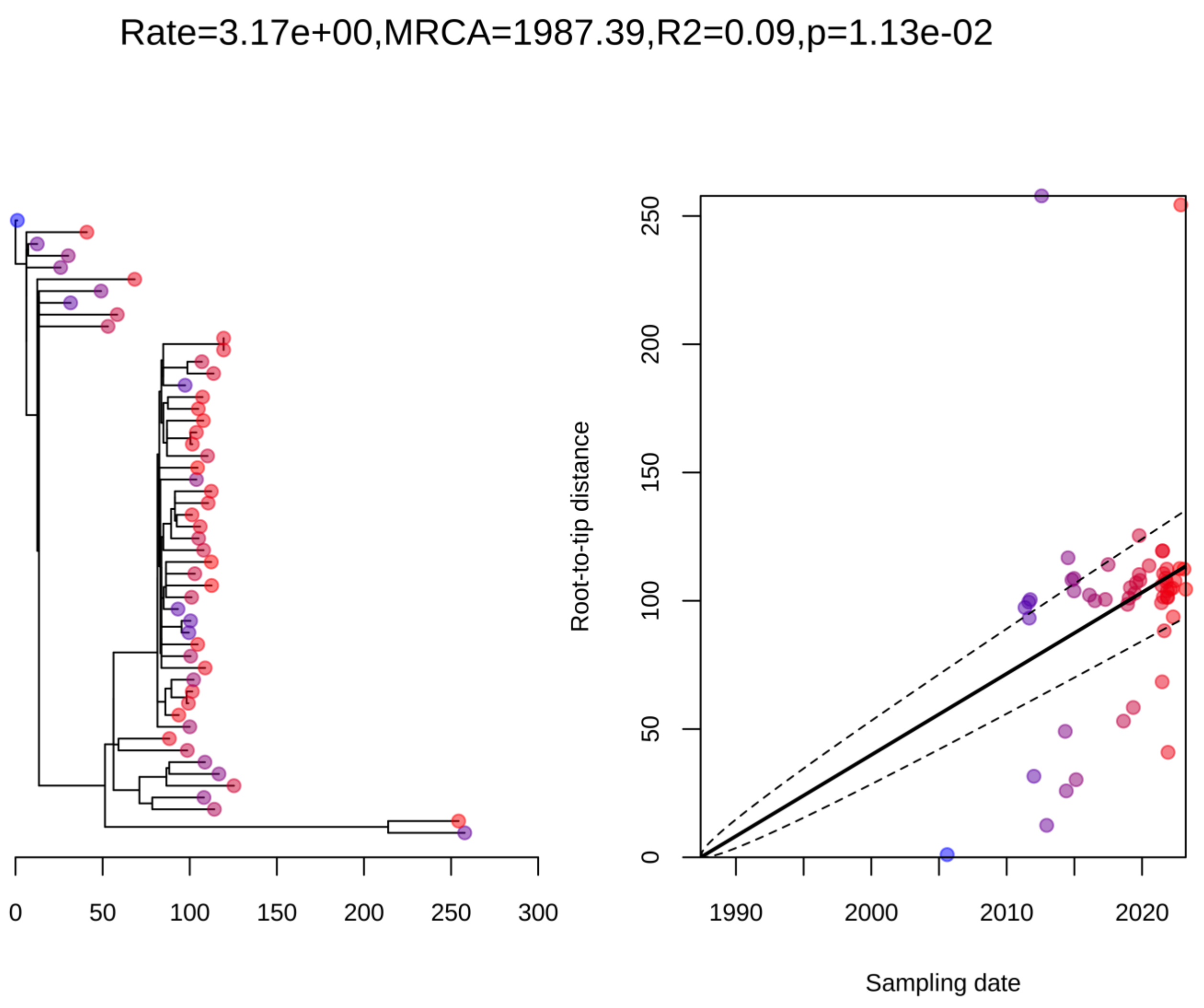

Figure S5. The results of root-to-tip linear regression analysis and permutation tests of the significance of the temporal signals using Bactdating. (a) ST10 (b) ST17 (c) ST23.

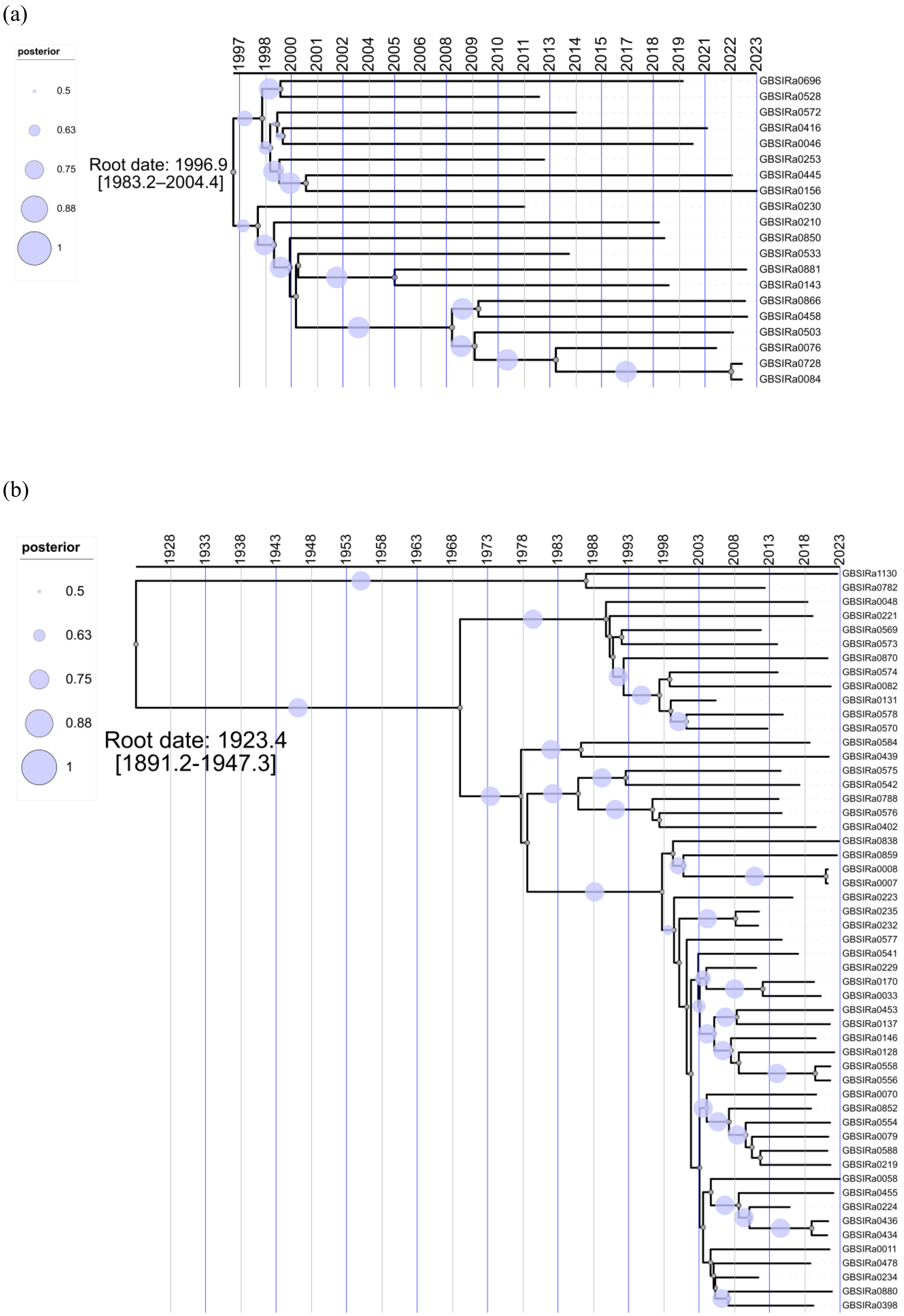

Figure S6. Phylogeny of date estimation analysis on ST10 and ST23 in Japan. Nodes of the phylogeny are placed at their median date. A root date is shown with 95% HPD intervals in each tree. (a) ST10 (b) ST23.

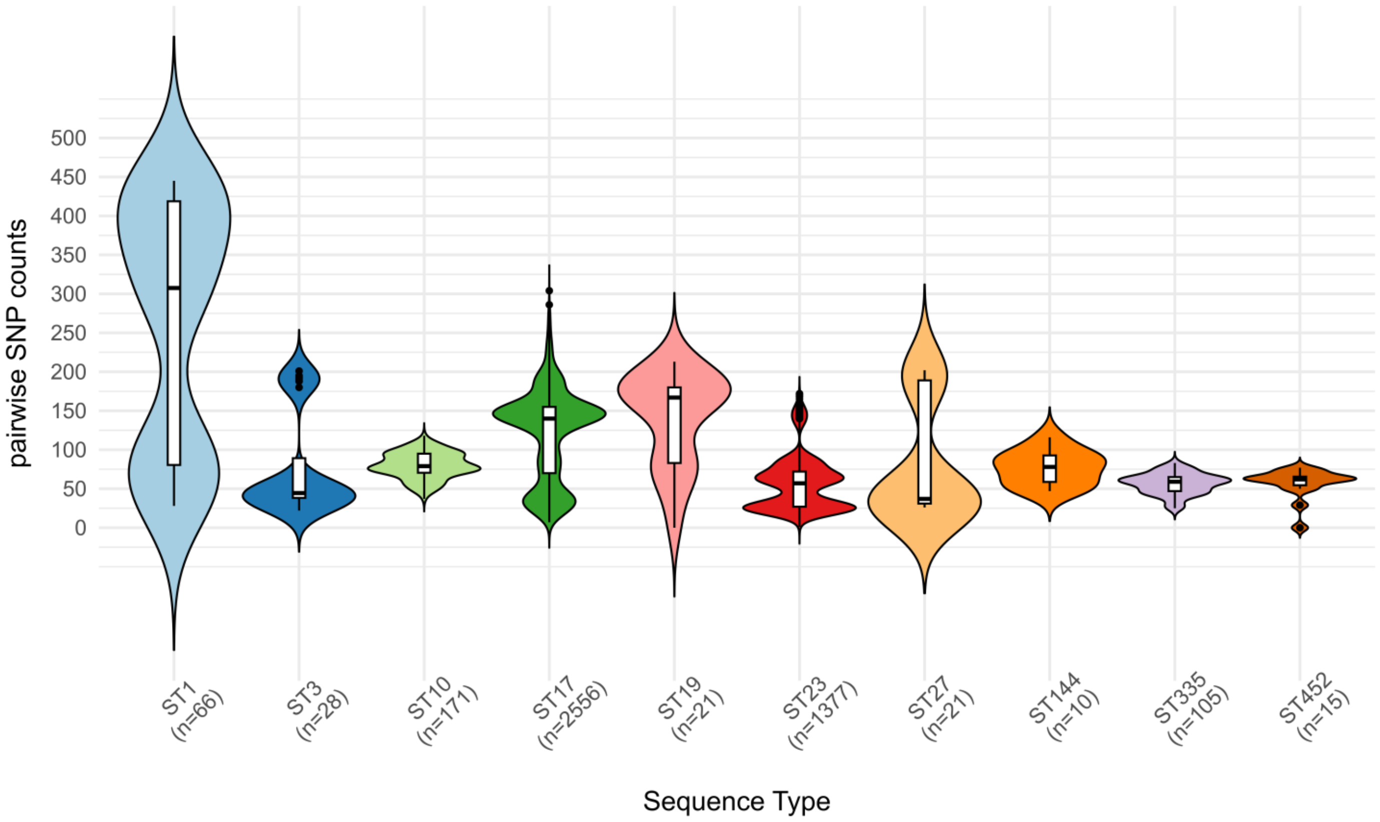

Figure S7. Violin plots of pairwise SNP distances among each ST with box plots. The number 'n' shown beneath each ST indicates the number of SNP distance observations, not the number of isolates. Boxplots are embedded within the violin plots. The black dots represent values that fall outside 1.5 times the interquartile range (IQR).

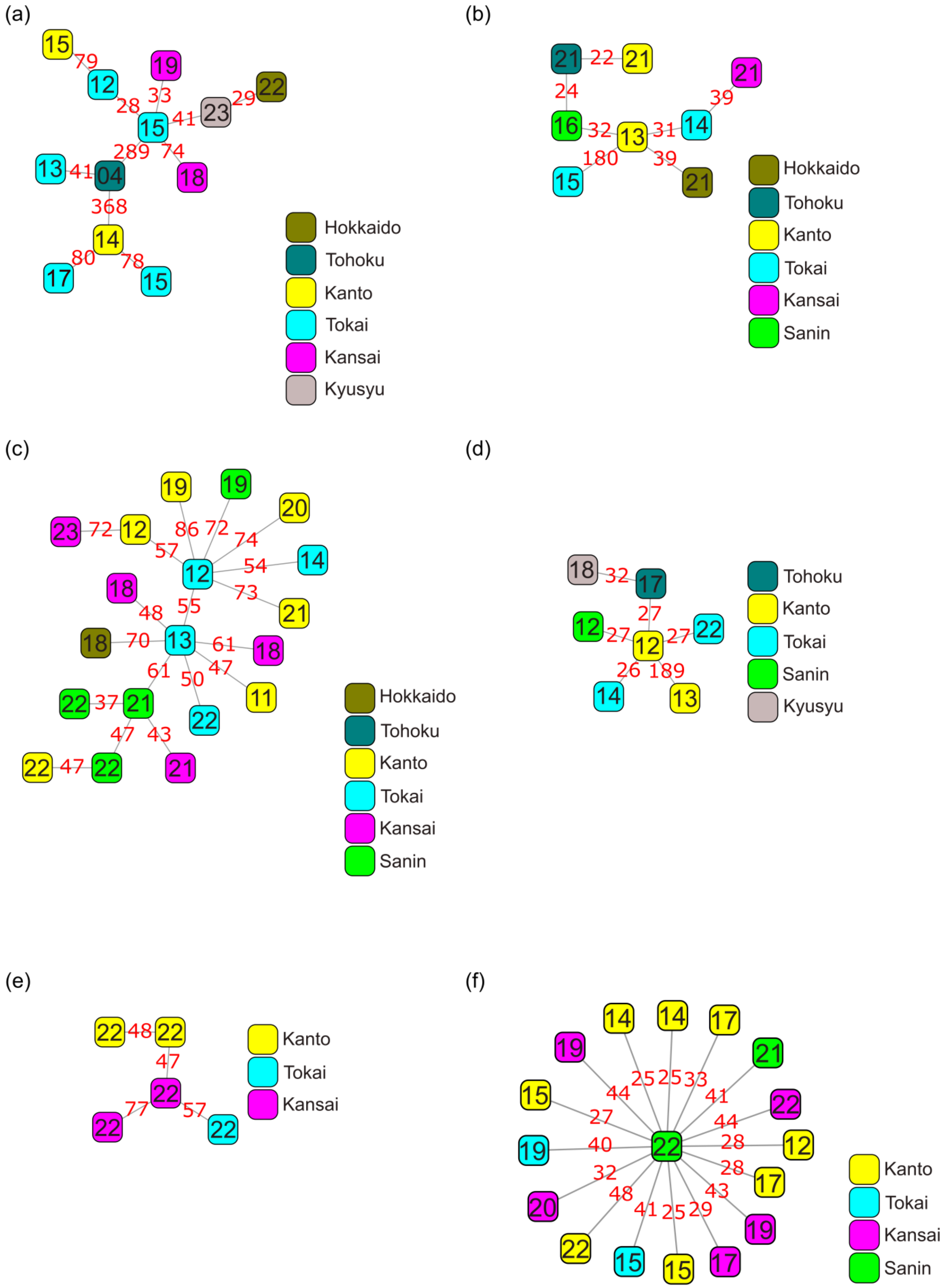
(a)
Hokkaido
Tohoku
Kanto
Tokai
Kansai
Kyusyu
(b)
Hokkaido
Tohoku
Kanto
Tokai
Kansai
Sanin
(c)
Hokkaido
Tohoku
Kanto
Tokai
Kansai
Sanin
(d)
Tohoku
Kanto
Tokai
Sanin
Kyusyu
(e)
Kanto
Tokai
Kansai
(f)
Kanto
Tokai
Kansai
Sanin

Figure S8. Minimum spanning trees created by GraphSNP with the metadata of detection regions, isolation year, and SNP distance. Each square represents a single isolate, with the number inside indicating the year of detection (e.g., '22' denotes the year 2022), and the color of the square corresponding to the geographic region of the hospital from which the isolate was detected. For region names, refer to Figure S1. The red numbers shown above the lines connecting strains indicate the number of SNPs between the respective strains, as calculated using the CF-SAN SNP pipeline. (a) ST1 (b) ST3 (c) ST10 (d) ST27 (e) ST144 (f) ST335.

(a)

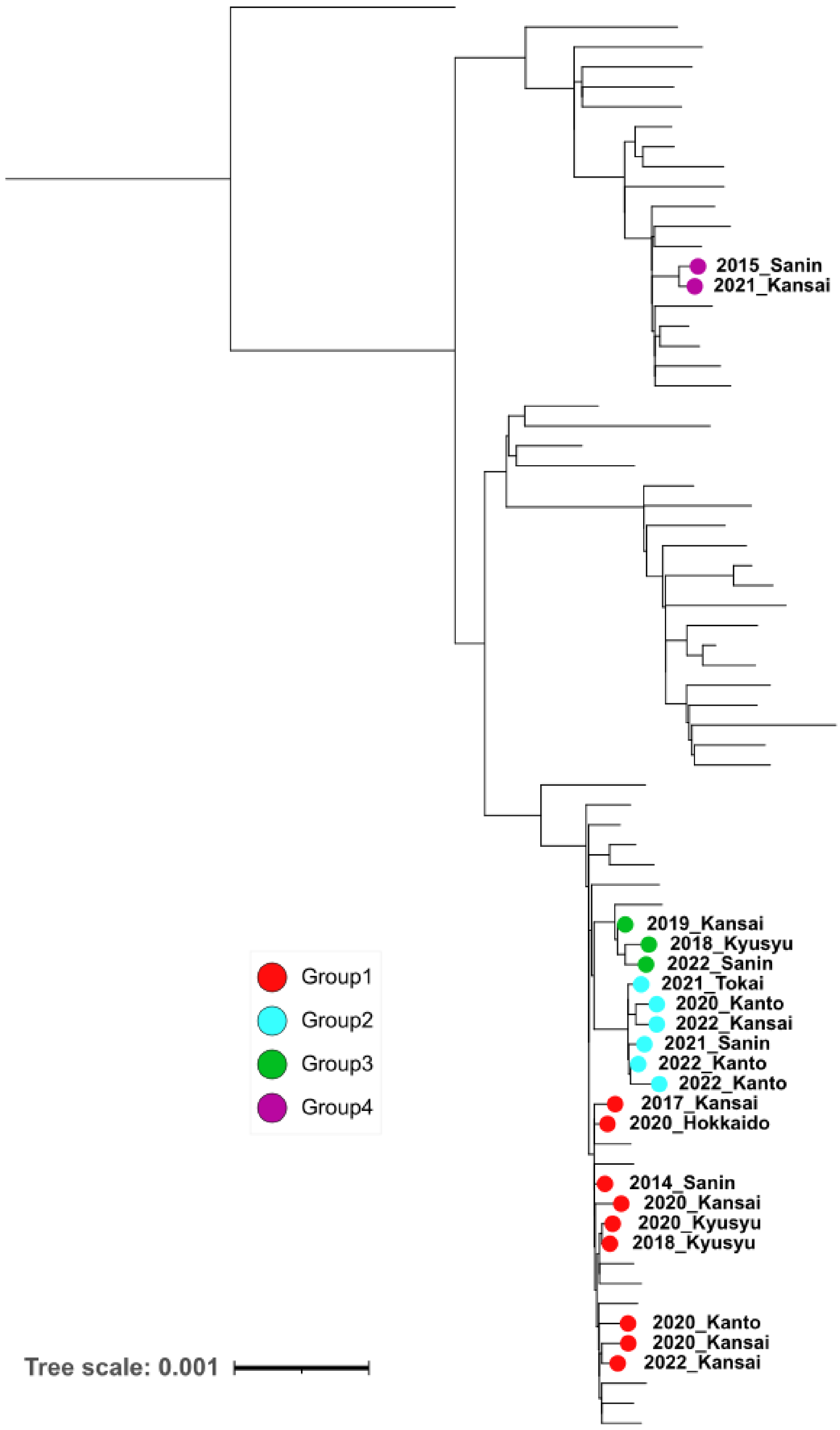
2015_Sanin
2021_Kansai
2019_Kansai
2018_Kyusyu
2022_Sanin
2021_Tokai
2020_Kanto
2022_Kansai
2021_Sanin
2022_Kanto
2022_Kanto
2017_Kansai
2020_Hokkaido
2014_Sanin
2020_Kansai
2020_Kyusyu
2018_Kyusyu
2020_Kanto
2020_Kansai
2022_Kansai
Group1
Group2
Group3
Group4
Tree scale: 0.001

(b)

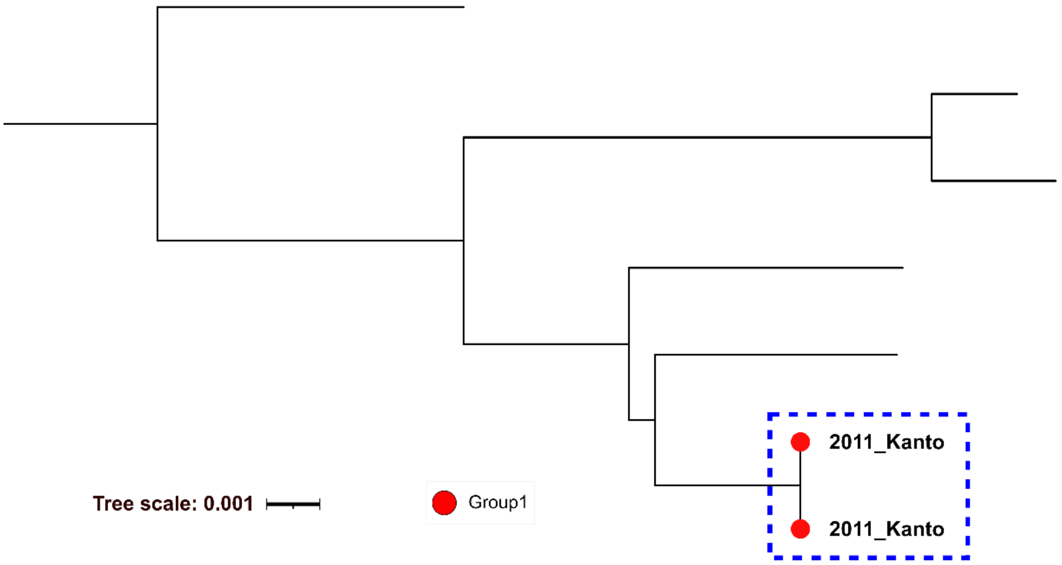
2011_Kanto
2011_Kanto
Tree scale: 0.001
Group1

(c)

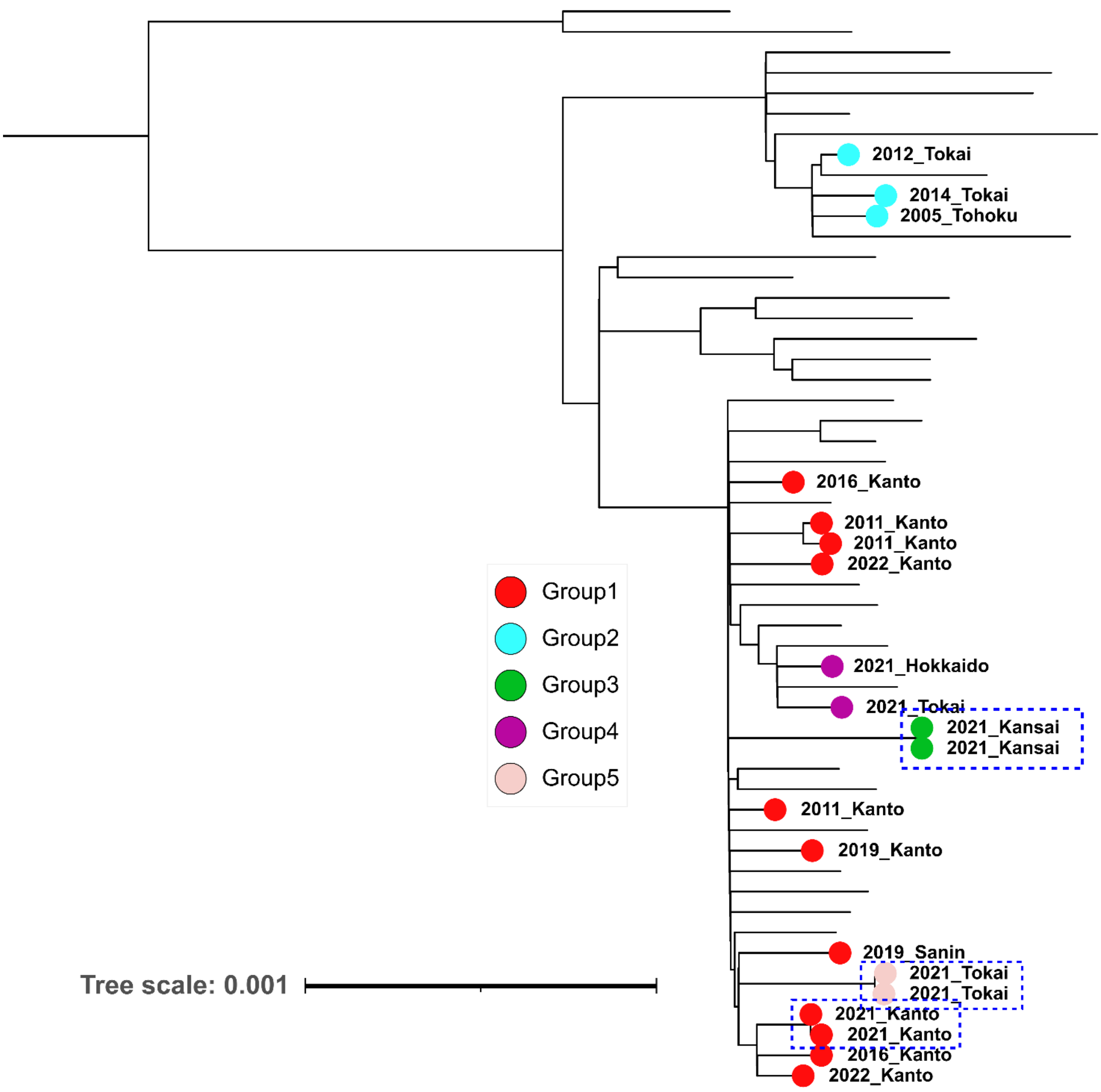
2012_Tokai
2014_Tokai
2005_Tohoku
2016_Kanto
2011_Kanto
2011_Kanto
2022_Kanto
Group1
Group2
Group3
Group4
Group5
2021_Hokkaido
2021_Tokai
2021_Kansai
2021_Kansai
2011_Kanto
2019_Kanto
2019_Sanin
2021_Tokai
2021_Tokai
2021_Kanto
2021_Kanto
2016_Kanto
2022_Kanto
Tree scale: 0.001

(d)

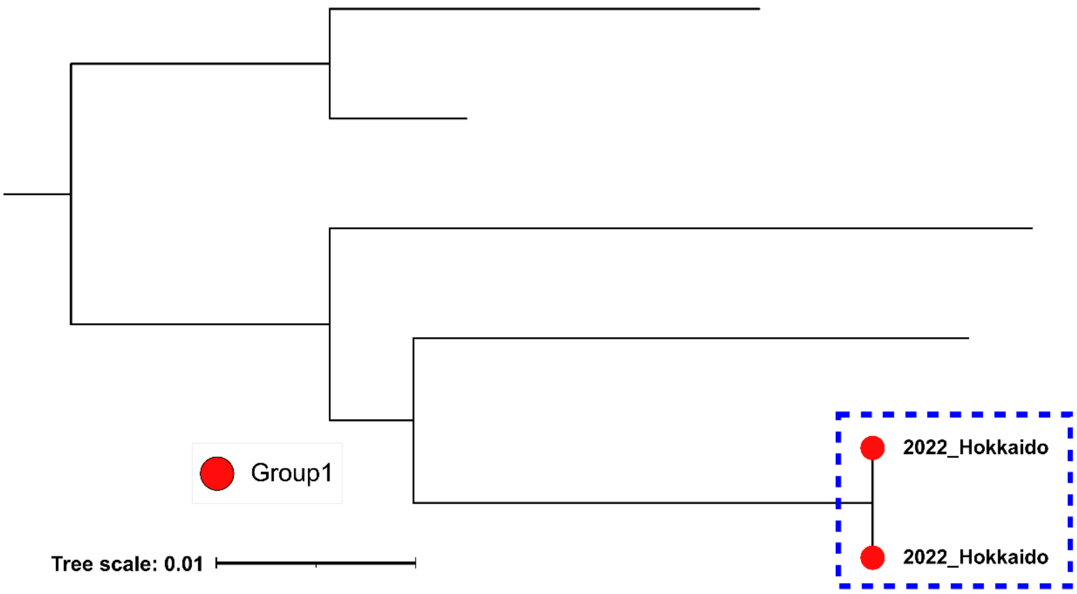

Figure S9. Maximum likelihood trees of each ST in which we detected endemic clusters and/or potential horizontal transmission. The group numbers indicated within each phylogenetic tree correspond to the group numbers assigned to each ST in the minimum spanning tree shown in Figure 2. The isolates enclosed by the blue dashed lines represent sets of isolates with a pairwise SNP distance of ≤4, both of which were recovered from the same hospital. (a) ST17 (b) ST19 (c) ST23 (d) ST452.

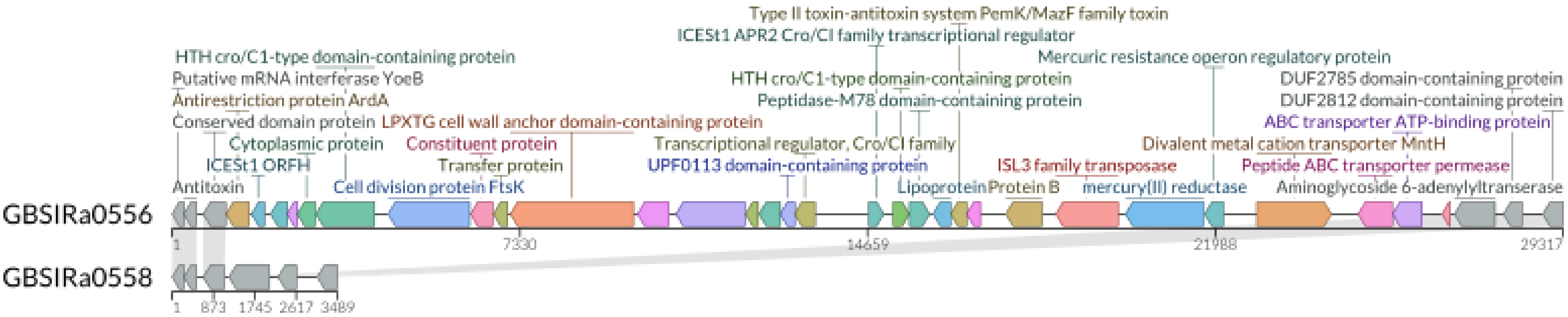

Figure S10. Genome comparison of two isolates detected in the same hospital with an SNP distance of 4, in which direct transmission was suspected. As shown in the figure, this region contained an indel of approximately 25.2 kb.

New PBP2X sequences

>GBSIRa0060(III-ST464/CC452)_GBSIRa0316(III-ST464/CC452)
GSTMKVMTLASAIDSKHFNSTEVYNSAQYKIADAVIRDWDVNEGLSSGSYMTFPQAFAHSSNVGM
VTLELKMGRDKWLNYLSKFKFGYPTRFGMLYESGGLFPSDNEVTIAMSSFGQGIGVTQVQMLRAF
TSISNDGVMLQPQFISSIYDPNTGTSRTARKEVVGKPVSKEAASKTRDYMVTVGTDPYYGTLYAAG
APVIQVGNQSVAVKSGTAQIAQEGGGGYLQGKNDTINSVVAMVPSENPDFIMYVTIQQPEKFSITFW
KDVVNPVLEQATAMKETILKPGLNDSEHQTKYKLSKIVGENPGHVAEELRRNLVQPIILGNGSKVSK
VSKRPGANLAENEQLL

>GBSIRa0152(Ib-ST2309/CC12)
GSTMKVMTLASAIDSKHFNSTEVYNSAQYKIADAIIRDWDVNEGLSSGSYMTFPQGFAHSSNVGM
VTLEQKMGRDKWLNYLSKFKFGYPTRFGMLHESGGLFPSDNEVTIAMSSFGQGIGVTQVQMLRAF
TSISNDGVMLQPQFISSIYDPNTGTSRTARKEVVGKPVSKEAASKTRDYMVTVGTDPYYGTLYAAG
APVIQVGNQSVAVKSGTAQIAQGGGGGYLQGKNDTINSVVAMVPSENPDFIMYVTIQQPEKFSITFW
KDVVNPVLEQATAMKETILKPGLNDSEHQTKYKLSKIVGENPGHVAEELRRNLVQPIILGNGSKVSK
VSKRPGANLAENEQLL

>GBSIRa0574(Ia-ST23/CC23)
GSTMKVMTLASAIDSKHFNSTEVYNSAQYKIADAVIRDWDVNEGLSSGSYMTFPQGFAHSSNVVM
VTLEQKMGRDKWLNYLSKFKFGYPTRFGMLHESGGLFPSDNEVTIAMSSFGQGIGVTQVQMLRAF
TSISNDGVMLQPQFISSIYDPNTGTSRTARKEVVGKPISKEAASKTRDYMVTVGTDPYYGTLYAAGA
PVIQVGNQSVAVKSGTAQIAQEGGGGYLQGKNDTINSVVAMVPSENPDFIMYVTIQQPEKFSITFWK
DVVNPVLEQATAMKETILKPGLNDSEHQTKYKLSKIVGENPGHVAEELRRNLVQPIILGNGSKVSKV
SKRPGANLAENEQLL

Sequences of novel vaccine candidate proteins

>BibA

MNNNEKKVKYFLRKTAYGLASMSAAFIVCSGIVNTPTVSADSPDTLKVEKLGKLKDVKSVHELTPI
SIPNELKGAKEQALSSIISHPNITNSEVDKLASDYSFRINTSNDVNDVKRLLNEFYNAVARKQLDTNS
ADYRSKIDNISTTGLAIALEAKEIYEANKSILPHRYKDSVGTYVNSFEERRSPGKFNIWNGQEGFNAA
QKLLEDVKKLLLELQNLTKNNKPNIQVPKQAPTEAAKPALSPEALTRLTTWYNQAKDLLKDDQVK
DKYVDILSVQKAVDQAYDHVEEGKFITTDQANQLANKLRDALQSLELKDKKVAKPVAKGTYDVK
YVDTEGKEVAKSRHFEGEEGAAFVTSAKEVAGYKLVRTEGAVSNVFTAGAQVRTYVYEKVAKPVA
KGTYDVKYVDTEGKEVAKSRHFEGEEGAAFVTSAKEVAGYKLVRTEGAVSNVFTAGAQVRTYVYE
KVKPEVKPDVKPEAKPEAKPEVKPDVKPEAKPEAKPEVKSDVKPEAKPEAKPEAKPEVKPDVKPE
AKPEAKPATKKSVNTSGNLVAKKAIENKKYSKKLPSTGEAASPLLAIVSLIVMLSAGLITIVLKHKKN

>C5a peptidase

MRKKQKLPFDKLAIALISTSILLNAQSDIKANTVTEDTPATEQAVEPPQPIAVSEESRSSKETKTSQTPS
DVGETVADDANDLAPQAPAKTADTPATSKATIRDLNDPSHVKTLQEKAGKGAGTVVAVIDAGFDKN
HEAWRLTDKTKARYQSKENLEKAKKEHGITYGEWVNDKVAYYHDYSKDGKNAVDQEHGTHVSGI
LSGNAPSEMKEPYRLEGAMPEAQLLLMRVEIVNGLADYARNYAQAIRDAVNLGAKVINMSFGNAA
LAYANLPDETKKAFDYAKSKGVSIVTSAGNDSSFGGKPRLPLADHPDYGVVGTPAAADSTLTVASYS
PDKQLTETATVKTDDHQDKEMPVISTNRFEPNKAYDYAYANRGTKEDDFKDVEGKIALIERGDIDFK
DKIANAKKAGAVGVLIYDNQDKGFPIELPNVDQMPAAFISRRDGLLLKDNPPKTITFNATPKVLPTAS
GTKLSRFSSWGLTADGNIKPDIAAPGQDILSSVANNKYAKLSGTSMSAPLVAGIMGLLQKQYETQYP
DMTPSERLDLAKKVLMSSATALYDEDEKAYFSPRQQGAGAVDAKKASAATMYVTDKDNTSSKVHL
NNVSDKFEVTVTVHNKSDKPQELYYQVTVQTDKVDGKHFALAPKALYETSWQKITIPANSSKQVT
VPIDASRFSKDLLAQMKNGYFLEGFVRFKQDPTKEELMSIPYIGFRGDFGNLSALEKPIYDSKDGSS
YYHEANSDAKDQLDGDGLQFYALKNNFTALTTESNPWTIIKAVKEGVENIEDIESSEITETIFAGTFAK
QDDDSHYYIHRHANGKPYAAISPNGDGNRDYVQFQGTFLRNAKNLVAEVLDKEGNVVWTSEVTE
QVVKNYNNDLASTLGSTRFEKTRWDGKDKDGKVVANGTYTYRVRYTPISSGAKEQHTDFDVIVDN
TTPEVATSATFSTEDSRLTLASKPKTSQPVYRERIAYTYMDEDLPTTEYISPNEDGTFTLPEEAETMEG
ATVPLKMSDFTYVVEDMAGNITYTPVTKLLEGHSNKPEQDGSDQAPDKKPEAKPEQDGSGQTPDK
KKETKPEKDSSGQTPGKTPQKGQSSRTLEKRSSKRALATKASTRDQLPTTNDKDTNRLHLLKLVMT
TFFLG

>Enolase

MAIITDVYAREVLDSRGNPTLEVEVYTESGAFGRGMVPSGASTGEHEAVELRDGDKSRYGGLGTQK
AVDNVNNVIAEAIIGYDVRDQQAIDRAMIALDGTPNKGKLGANAILGVSIAVARAAADYLEVPLYS
YLGGFNTKVLPTPMMNIINGGSHSDAPIAFQEFMIMPVGAPTFKEALRWGAEVFHALKKILKERGL
ETAVGDEGGFAPKFEGTEDGVETILKAIEAAGYEAGENGIMIGFDCASSEFYDAERKVYDYSKFEGE
GGAVRTAAEQIDYLEELVNKYPIITIEDGMDENDWDGWKALTERLGGRVQLVGDDFFVTNTDYLAR
GIKEEAANSILIKVNQIGTLTETFEAIEMAKEAGYTAVVSHRSGETEDSTIADIAVATNAGQIKTGSLSR
TDRIAKYNQLLRIEDQLGEVAQYKGIKSFYNLDKCGR

>Glyceraldehyde-3-phosphate dehydrogenase

MVVKVGINGFGRIGRLAFRRIQNVEGVEVTRINDLTDPNMLAHLLKYDTTQGRFDGTVEVKEGGFE
VNGQFVKVSAEREPANIDWATDGVEIVLEATGFFASKEKAEQHIHENGAKKVVITAPGGNDVKTVV
FNTNHDILDGTETVISGASCTTNCLAPMAKALQDNFGVKQGLMTTIHAYTGDQMILDGPHRGGDL

RRARAGAANIVPNSTGAAKAIGLVIPELNGKLDGAAQRVPVPTGSVTELVATLEKDVTVEEVNAAM
KAAANDSYGYTEDPIVSSDIVGISYGSLFDATQTKVQTVDGNQLVKVVSWYDNEMSYTSQLVRTLE
YFAKIAK
>gbs2106
ADKVRVAKKSKMTKATSKSKVEDVKQAPKPSQASNEAPKSSSQSTEANSQQQVTASEEAAVEQAV
VTENTPATSQAQQAYAVTETTYRPAQHQTSGQVLSNGNTAGAIGSAAAAQMAAATGVPQSTWEHII
ARESNGNPNVANASGASGLFQTMPGWGSTATVQDQVNSAIKAYRAQGLSAW
>LrrG
MTKKHLKTLALALTTVSVVTYSQEVYGLEREESVKQEQTQSASEDDWFEEDNERKTNVSKENSTV
DETVSDLFSDGNSNNSSSKTESVVSDPKQVPKAKPEVTQEASNSSNDASKVEVPKQDTASKKETLE
TSTWEAKDFVTRGDTLVGFSKSGINKLSQTSHLVLPSHAADGTQLTQVASFAFTPDKKTAIAEYTSRL
GENGKPSRLDIDQKEIIDEGEIFNAYQLTKLTIPNGYKSIGQDAFVDNKNIAEVNLPESLETISDYAFAH
MSLKQVKLPDNLKVIGELAFFDNQIGGKLYLPRHLIKLAERAFKSNRIQTVEFLGSKLKVIGEASFQD
NNLRNVMLPDGLEKIESEAFTGNPGDEHYNNQVVLRTRTGQNPHQLATENTYVNPDKSLWRATPD
MDYTKWLEEDFTYQKNSVTGFSNKGLQKVRRNKNLEIPKQHNGITITEIGDNAFRNVDFQSKTLRK
YDLEEIKLPSTIRKIGAFAFQSNNLKSFEASEDLEEIKEGAFMNNRIGTLDLKDKLIKIGDAAFHINHIY
AIVLPESVQEIGRSAFRQNGALHLMFIGNKVKTIGEMAFLSNKLESVNLSEQKQLKTIEVQAFSDNA
LSEVVLPPNLQTIREEAFKRNHLKEVKGSSTLSQITFNAFDQNDGDKRFGKKVVVRTHNNSHMLAD
GERFIIDPDKLSSTMVDLEKVLKIIEGLDYSTLRQTTQTQFREMTTAGKALLSKSNLRQGEKQKFLQ
EAQFFLGRVDLDKAIAKAEKALVTKKATKNGHLLERSINKAVLAYNNSAIKKANVKRLEKELDLLT
DLVEGKGPLAQATMVQGVYLLKTPLPLPEYYIGLNVYFDKSGKLIYALDMSDTIGEGQKDAYGNPI
LNVDEDNEGYHTLAVATLADYEGLYIKDILNSSLDKIKAIRQIPLAKYHRLGIFQAIRNAAAEADRLL
PKTPKGYLNEVPNYRKKQMEKNLKPVDYKTPIFNKALPNEKVDGDRAAKGHNINAETNNSVAVTPI
RSEQQLHKSQSDVNLPQTSSKNNFIYEILGYVSLCLLFLVTAGKKGKRARK
>SIP
MKMNKKVLLTSTMAASLLSVASVQAQETDTTWTARTVSEVKADLVKQDNKSSYTVKYGDTLSVIS
EAMSIDMNVLAKINNIADINLIYPETTLTVTYDQKSHTATSMKIETPATNAAGQTTATVDLKTNQVSV
ADQKVSLNTISEGMTPEAATTIVSPMKTYSSAPALKSKEVLAQEQAVSQAAANEQVSPAPVKSITSE
VPAAKEEVKPTQTSVSQSTTVSPASVAAETPAPVAKVAPVRTVAAPRVASVKVVTPKVETGASPEHV
SAPAVPVTTTSPATDSKLQATEVKSVPVAQKAPTATPVAQPASTTNAVAAHPENAGLQPHVAAYKEK
VASTYGVNEFSTYRAGDPGDHGKGLAVDFIVGTNQALGNKVAQYSTQNMAANNISYVIWQQKFYS
NTNSIYGPANTWNAMPDRGGVTANHYDHVHVSFNK
>SAN_0226
NQNSQTKERTRKQRPKDELVVSMGAKLPHEFDPKDRYGIHNEGNITHSTLLKRSPELDIKGELAKK
YKISKDGLTWSFDLNDDFKFSNGEPVTADDVKFTYDMLKADGKAWDLTFIKNVEVVGKNQVNIHL
TEAHSTFTAQLTEIPIVPKKHYNDKYKSNPIGSGPYMVKEYKAGEQAIFVRNPYWHGKKPYFKKWT
WVLLDENTALAALESGDVDMIYATPELASKKVKGTRLLDIASNDVRGLSLPYVKKGVVKNSPDGY
PVGNDVTSDPAIRKALTIGLNRQKVLDTVLNGYGKPAYSIIDRTPFWNPKTAIKDNKVAKAKQLLTK
AGWKEQADGSRKKGNLKAEFDLYYPTNDQLRANLAVEVAEQAKALSITIKLKASNWDEMATKSH
DSALLYAGGRHHAQQFYESHYPSLAGKGWTNITFYNNPTVTKYLDKAMTSPDLDKANKYWKLAQ
WDGKTGASTLGDLPNVWLVSLNHTYIGDKRINVGKQGVHSHGHDWSLLTNIAEWTWDESAK
>SAN_0356

KILVSPTTIRVPDVSNKTVAQAKMTLENSGLKVGAIRNIESDSVSEGLVVKTDPAAGRSRREGAKVN
LYIATPNKSFTLGNYKEHNYKDILKDLQGKGVKKSLIKVKRKINNDYTTGTILAQSLPEGTSFNPDG
NKKLTLTVAVNDPMIMPDVTGMTVGEVIETLTDLGLDADNLVFYQMQNGVYQAVVTPPSSSKIASQ
DPYYGGEVGLRRGDKVKLYLLGSKTTNNSSSTPIDSSASSSTGTTTSDSVSSSTDASTSDSSSTSTSSS
TLPSDSTTNTGTANNPLTQ
>SAN_0413
YNFSTNELSKTFKDFKLAKSKSHAIEETKPFSILLMGVDTGSEHRKSKWSGNSDSMILVTINPKTNKT
TMTSLERDVLIKLSGPKNNGQTGVEAKLNAAYASGGAEMALMTVQDLLDINVDYFMQINMQGLV
DLVNAVGGITVTNKFDFPISIAANEPEYKAVVEPGTHKINGEQALVYSRMRYDDPEGDYGRQKRQR
EVIQKVLKKILALNSISSYKKILSAVSNNMQTNIEISSKTIPNLLAYKDSLEHIKSYQLKGEDATLSDG
GSYQILTKKHLLAVQNRIKKELDKKRSKTLKTSAILYEDYYGTTASNDSSTYSSTQENNYNTTPYSE
APPSYSGNTTYSSETNQTTHQSYYNSSTPASNYSSNTNTGQADSSGSVNNYNGAATPNPNTGTQPVP
SQTNP
>SAN_0990
NHQDNSKIAGASETYTHTLTDVPIDIKYDSDDYFISGYSYGADVYMSSVNRVKLDSEINEDTRKFKV
VADLTNMKPGTHKVPLKVVNLPSGVNATVSPTTITVTMGKKKTKEFPVYGHVNDKQIKAGYAVDK
MSVDVSKVKVTSDESIIDRIDHVAANIPDDKVLDDDFNKTVTLQAVTADGTVLASIIHPSKATLSVKV
KKLTKTVPINLIPVGQFSDSISKINYKLSQEKAVISGTKEALEAISVINAEVDISDVTKNTEKKINLSAN
NVSVDPAQVTVQLTTTKK
>SAN_1040
SGGKSDSLKVAMVTDTGGVDDKSFNQSGWEGMQAWGKKNGLKKGAGFDYFQSASESDYATNLD
TAVSSGYKLIFGIGFSLHDAIDKAADNNKDVNYVIVDDVIKGKDNVASVVFADNESAYLAGIAAAKT
TKTKTVGFVGGMESEVITRFEKGFEAGVKSVDKSIKIKVDYAGSFGDAAKGKTIAAAQYASGADIV
YQVAGGTGAGVFSEAKSRNESLKEADKVWVLGVDRDQAAEGKYTSKDGKASNFVLASSIKEVGK
SVELIATKTSKGKFPGGNVTTYGLKDGGVDIATTNLSDDAVKAIKEAKAKIISGDIKVPSK
>SAN_1577
ADTSDKNTDTSVVTTTLSEEKRSDELDQSSTGSSSENESSSSSEPETNPSTNPPTTEPSQPSPSEENKP
DGSTKTEIGNNKDISSGTKVLISEDSIKNFSKASSDQEEVERDESSSSKANDEKKGHSKPKKELPKTG
DSHSD
>SAN_1685
STGTKIGKDIKVGYNWELSGNVSSYGNSMKNGADLAVKEINAAGGVGGKKLKVLSQDNKSENAE
AATVATNLVTKGANVIIGPATSGAAASSTPKVNAAAVPMIAPAATQDNLVYGSDGKTLNQYFFRATF
VDNYQGKLLSQYATDNLKAKKVVLFYDNSSDYSKGVAKSFKESYSGKIVDSMTFSAGDTDFQASLT
KLKGKEYDAIVMPGYYTETGLIVKQARDLGISKPVLGPDGFDSPKFVQSATPVGASNVYYLTGFTT
QGSTKAKAFHDHYVKAYGEEPSMFSALSYDAVYMAAKSAKGAKTSIDIKKALAKLKDFKGVTGK
MSIDKNHNVVKSAYVVKLEDGKTSSVNIISAK
>SAN_1808
ETINPETSLTMATASTESSSEAEKQEKTQPTDSETASPSAEGSISTEKTEIGTTETSSSNESSSSSSHQSSS
NEDAKTSDSASTASTPSTNTTNSSQADSKPGQSTKTELKPEPTLPLVEPKITPAPSQIESVQTNQNASV
PALSFDDNLLSTPISPVTATPFYVEHWSGQDAYSHYLLSHRYGIKAEQLDGYLKSLGIQYDSNRINGA
KLLQWEKDSGLDVRAIVAIAVLESSLGTQGVAKMPGANMFGYGAFDHDSSHASAYNDEEAIMLLT
KNTIIKNNNSSFEIQDLKAQKLSSGQLNTVTEGGVYYTDNSGTGKRRAQIMEDLDRWIDQHGGTPEI

PAALKALSTASLADLPSGFSLSTAVNTASYIASTYPWGECTWYVFNRAKELGYTFDPFMGNGGDWQ
HKAGFETTHSPKVGYAVSFSPGQAGADGTYGHVAIVEEVKKDGSVLISESNAMGRGIVSYRTFSSAQ
AAQLTYVIGHK
